# Translating the *Arabidopsis thaliana* Peroxisome Proteome Insights to *Solanum lycopersicum*: Consensus Versus Diversity

**DOI:** 10.3389/fcell.2022.909604

**Published:** 2022-07-13

**Authors:** Sabiha Tarafdar, Gopal Chowdhary

**Affiliations:** Plant Molecular Biology Laboratory, School of Biotechnology, KIIT, Bhubaneswar, India

**Keywords:** *Solanum lycopersicum*, peroxisome, *Arabidopsis thaliana*, peroxisome targeting signal, canonical and non-canonical signal

## Abstract

Peroxisomes are small, single-membrane specialized organelles present in all eukaryotic organisms. The peroxisome is one of the nodal centers of reactive oxygen species homeostasis in plants, which are generated in a high amount due to various stress conditions. Over the past decade, there has been extensive study on peroxisomal proteins and their signaling pathways in the model plant Arabidopsis thaliana, and a lot has been deciphered. However, not much impetus has been given to studying the peroxisome proteome of economically important crops. Owing to the significance of peroxisomes in the physiology of plants during normal and stress conditions, understating its proteome is of much importance. Hence, in this paper, we have made a snapshot of putative peroxisomal matrix proteins in the economically important vegetable crop tomato (Solanum lycopersicum, (L.) family Solanaceae). First, a reference peroxisomal matrix proteome map was generated for Arabidopsis thaliana using the available proteomic and localization studies, and proteins were categorized into various groups as per their annotations. This was used to create the putative peroxisomal matrix proteome map for S. lycopersicum. The putative peroxisome proteome in S. lycopersicum retains the basic framework: the bulk of proteins had peroxisomal targeting signal (PTS) type 1, a minor group had PTS2, and the catalase family retained its characteristic internal PTS. Apart from these, a considerable number of S. lycopersicum orthologs did not contain any “obvious” PTS. The number of PTS2 isoforms was found to be reduced in S. lycopersicum. We further investigated the PTS1s in the case of both the plant species and generated a pattern for canonical and non-canonical PTS1s. The number of canonical PTS1 proteins was comparatively lesser in S. lycopersicum. The non-canonical PTS1s were found to be comparable in both the plant species; however, S. lycopersicum showed greater diversity in the composition of the signal tripeptide. Finally, we have tried to address the lacunas and probable strategies to fill those gaps.

## Introduction

In the course of evolution, the eukaryotic cells have developed various compartments delimited by either a single or double membrane. Peroxisomes are one such single-membrane-bounded subcellular organelle. They have been shown to be of varying sizes, shapes, numbers, and protein contents depending on the developmental stage and the habitat in which the organism lives. They have also been shown to interact with other cell organelles like mitochondria, chloroplast, and endoplasmic reticulum and form intracellular signaling platforms (Schrader et al., 2013; Shai et al., 2016; Mast et al., 2018; Shai et al., 2018). Defects in peroxisome dynamics can lead to organelle dysfunction and have been associated with various disorders as well (Fransen, 2012; Deori et al., 2018). Peroxisomes exist in all eukaryotes from microorganisms to macroorganisms and to plants and animals.

When compared to nuclei, mitochondria, and chloroplasts, peroxisomes lack DNA. Therefore, all their proteins are encoded by the nuclear DNA, synthesized on cytosolic ribosomes, and post-translationally, fully folded proteins enter the organelle. The protein complement of peroxisome can be broadly divided into two groups – membrane protein and matrix protein (Sacksteder and Gould, 2000). In this paper, we will primarily concentrate on the peroxisomal matrix proteins. The protein transport to the peroxisomal matrix primarily depends upon the peroxisome targeting signals, present either at the C-terminus or at the N-terminus of the protein (Emmanouilidis et al., 2016). Evidence suggests that peroxisomes have originated from endosymbionts that subsequently lost their DNA; however, no definitive proof has yet been uncovered (Farré and Subramani, 2004).

Since the discovery of peroxisomes by de Duve in 1966 (De Duve, 1969), the list of physiological and metabolic functions related to peroxisomes has grown tremendously. However, the beta-oxidation of fatty acids and removal of hydrogen peroxide remain central to their metabolic role. In mammalian cells, the fatty acid breakdown pathway is distributed between peroxisomes and mitochondria, while that in plant and fungal cells is exclusively localized in peroxisomes. In trypanosomes, the part of glycolysis has also been reported to be localized in glycosomes (peroxisome-related organelle in kinetoplastids and diplonemids, Quiñones et al., 2020). Photorespiration has been deemed characteristic of plant peroxisomes; however, yeast peroxisomes can also oxidize alkanes or methanol (Subramani, 1998; Platta and Erdmann, 2007; Kaur and Hu, 2011). With the advent of modern techniques and newer research, the metabolic portfolio of peroxisomes now includes various aspects of lipid metabolism, purines, polyamines, amino acid catabolism, biosynthesis of jasmonic acid, indole 3-butyric acid (IBA), glyoxylate metabolism, the homeostasis of reactive oxygen species (ROS) (Fukao et al., 2003; Farré and Subramani, 2004; Baker and Sparkes, 2005; Reumann and Weber, 2006; Kaur et al., 2009; Palma et al., 2009; Kaur and Hu, 2011), production of glycine betaine, degradation of branched amino acids (Reumann, 2004), and the presence of regulatory proteins such as kinases, phosphatases, and heat-shock proteins as well (Hayashi and Nishimura, 2006; Reumann et al., 2007; Kataya et al., 2019). Lately, peroxisomes have also been found to be involved in biotic and abiotic stress acclimation as well (Choudhury et al., 2017). However, the primary metabolic profile of peroxisomes remains to be of the oxidative type (del Río et al., 2006; Fransen, 2012). Further, in a plant cell, the energy metabolic process is primarily distributed among the three main organelles of the chloroplast, mitochondria, and peroxisomes. These three organelles also form the tri-nodal center for cellular reactive oxygen species homeostasis in plant cells. The proteome complement of the chloroplast and mitochondria are well described and characterized, while that of peroxisomes is still lacking. The major bottleneck in this is the lack of availability of purified organelles for mass-spectrometry (MS) analysis. This problem is further compounded by the presence of peroxisomes in very less quantities in plants and their fragile nature. However, the lack of wet lab data sometimes may be complemented by *in silico* approaches, and hence multiple peroxisome targeting algorithms are being developed (Imai and Nakai, 2010; Reumann, 2011). *Arabidopsis* being a model plant has been put under investigation for peroxisome proteome in great detail (Fukao et al., 2002; Fukao et al., 2003; Reumann et al., 2007; Eubel et al., 2008; Quan et al., 2013). The major proteins present in *Arabidopsis* peroxisome have been described; however, the complete and holistic make-up of *Arabidopsis* peroxisome proteome is yet far from complete. Despite all its drawbacks, the *Arabidopsis* peroxisome proteome is the closest available peroxisome proteome for comparative analysis.

In this review, we have used *Arabidopsis* peroxisome proteome as a reference and the data obtained from the *Arabidopsis* have been used to deduce and predict the tentative peroxisome proteome list from tomato (*Solanum lycopersicum L.*) via *in silico* approaches, and the comparative account has been presented. Tomato is a vegetable crop of high economic and nutritional value. The tomato genome has also been recently sequenced and annotated (Sato et al., 2012). High-throughput proteomic and post-genomic analyses have also been performed to gain insights into various molecular networks (Kilambi et al., 2016; Liu et al., 2016; Mata et al., 2017). Understanding the metabolic, proteomic, and regulatory networks in these “minor” but vital organelles in important vegetable crops will be highly beneficial for agriculture in the coming days.

### Peroxisomal Targeting Signals

The assembly of functional peroxisomes requires the import of a large number of different nuclear-encoded proteins. These proteins can reside in the peroxisomal membrane or be confined to the matrix of the organelle. Since the peroxisomal proteins are synthesized on cytosolic ribosomes, they are dependent on peroxisome targeting signals (PTS) to be imported to peroxisomes. In general, two types of peroxisomal targeting signals are known: peroxisomal targeting signal (PTS) type 1 and type 2 (Walter and Erdmann, 2019). PTS1 is present at the C-terminus of protein, consisting of three terminal amino acids and upstream residues acting as enhancer elements, while PTS2 is present at the N-terminus and is represented by nine amino acids. Apart from PTS1 and PTS2, some non-conventional targeting systems such as piggybacking and internal PTS are also present in selected proteins (Glover et al., 1994; Lee et al., 1997; Yang et al., 2001; Titorenko et al., 2002; Johnson and Olsen, 2003; Kamigaki et al., 2003; Oshima et al., 2008; Wolf et al., 2010; Effelsberg et al., 2015; Kataya et al., 2015; Gabay-Maskit et al., 2020).

### Peroxisomal Targeting Signal Type 1 (PTS1)

Primarily, PTS type 1 proteins form the bulk of peroxisomal matrix proteins amounting to about 65% (Brocard and Hartig, 2006). The PTS1 tripeptide could be canonical and non-canonical. The canonical PTS1 leads to strong targeting of reporter proteins and usually has a consensus sequence of [SA] [KR], and [LMI] > at -3, -2, and -1 positions, respectively. These have been found to occur frequently in the peroxisomal matrix proteins of higher plants and hence considered to be of high abundance. The strong targeting signifies that the reporter proteins were detectable in peroxisomes within 24 h post-transformation under *in vitro* studies (Skoulding et al., 2015).

The non-canonical type tripeptides contain one non-canonical residue (a low-abundance residue) at any of the tripeptide positions, for example, ASL>, SLM (underlined residues represent the non-canonical or low-abundance type), etc. Nearly all the non-canonical PTS1s identified to date follow this pattern of having one low-abundance (non-canonical) and two high-abundance (canonical) residues in the tripeptide positions. However, Skoulding et al. (2015) identified one tripeptide to be functional PTS1 as SNV> (”>” symbol denotes the end of the polypeptide chain) where two amino acid residues, asparagine and valine, at -2 and -1 positions, respectively, were found to be non-canonical or of low abundance in nature. With time, more experimental data are made available, more elaboration on non-canonical PTS1 tripeptides could be made. Due to the lack of sufficient raw data, it is difficult to computationally identify the non-canonical PTS1 tripeptides (Lingner et al., 2011; Chowdhary et al., 2012).

PTS1 is recognized by the PEX5 receptor in the cytosol which binds directly to the PTS1 tripeptide. The intensity of interaction between PEX5 and different PTS1 signals differs among species (Lametschwandtner et al., 1998). In the case of canonical PTS1, where there is binding of peroxisomal protein to its cytosolic receptor PEX5, the primary and significant role is played by the C-terminus tripeptide, while the upstream residues have also been shown to exert a minor effect on the PEX5 binding (Lametschwandtner et al., 1998; Neuberger et al., 2003; Reumann 2004; Brocard and Hartig 2006; Lingner et al., 2011; Fodor et al., 2012). In the case of non-canonical PTS1, the upstream residues play a significant role in PEX5 binding (Reumann et al., 2016). The binding of the PTS1 cargo and PEX5 is a dynamic one. The terminal carboxylate group of the PTS1 cargo binds to the N415, N526, and R520 of PEX5 (in the human variant of PEX5). The peptide backbone of the PTS1 cargo binds to N561. The side chain of the PTS1 cargo binds to the pockets present in PEX5 and provides specificity. The cargo binding to PEX5 leads to a change in the conformation of PEX5 from an open conformation to a closed ring-like conformation (Gatto et al., 2000; Stanley et al., 2006), suggesting that the ligand binding induces the conformation change (Zeytuni et al., 2011; Fodor et al., 2015). The conformational flexibility of PEX5 is the key, which explains its binding ability with a varied number of amino acids as its cargo.

Followed by PTS1-PEX5 binding, there lies an interaction with the docking peroxins PEX13 and PEX14 at the peroxisomal membrane (Albertini et al., 1997; Bottger et al., 2000; Niederhoff et al., 2005). After delivering the cargo, the PEX5 proteins are recycled back to the cytosol in an ATP-dependent manner (Kim and Hettema, 2015). Some organisms contain two forms of PEX5, a long form, PEX5L, and a short form, PEX5S. The long form contains an additional domain for PEX7 binding, thereby mediating the PTS2 import pathway as well. PEX5S is exclusively involved in PTS1 cargo import, while PEX5L is involved in both PTS1 and PTS2 cargo import (Lee et al., 2006). *Arabidopsis thaliana* contains only PEX5S, while *Oryza sativa* contains both long and short forms (Lee et al., 2006). The protein BLAST searches also revealed that *Solanum lycopersicum* also contains only the short form of PEX5.

### Peroxisomal Targeting Signal Type 2 (PTS2)

There is yet another subset of peroxisomal matrix proteins that is mediated by a type 2 peroxisomal targeting signal (PTS2) (Lazarow, 2006). This signal is a degenerated nonapeptide and can be found in the N-terminal portion of a limited number of peroxisomal matrix proteins. PTS2 proteins are less abundant than PTS1 proteins. In some organisms, the number of PTS2 proteins has been reduced to 2 only, such as *Saccharomyces cerevisiae* (glycerol-3-phosphate dehydrogenase and 3-ketoacyl-CoA thiolase/Fox3p) and *Trypanosoma* (aldolase and thiolase) (Blattner et al., 1995). Certain organisms such as *Drosophila melanogaster* (Faust et al., 2012), *Caenorhabditis elegans* (Gurvitz et al., 2000; Motley et al., 2000), *Cyanidioschyzon merolae* (Misumi et al., 2005), and diatoms (Gonzalez et al., 2011) showed complete loss of the PTS2-import pathway and thereby rely solely on PTS1-dependent import.

The “consensus” sequence for PTS2 is ([RK][LVIQ]x2 [LVIHQ][LSGAK] × [HQ][LAF]) (Petriv et al., 2004). PTS2 protein is imported to the peroxisomal matrix via its cytosolic receptor PEX7. Due to the comparatively longer nature of the signal, it is difficult to derive a consensus motif in the case of PTS2. The PTS2 motif contains amino acids with helix-forming propensity, suggesting that the motif could form a helix-like structure that gets inserted into its cytosolic receptor PEX7 (Reumann 2004; Kiel et al., 2009; Kunze et al., 2011). The crystal structure of yeast thiolase (Fox3p) bounded to PEX7 has also revealed the same (Pan et al., 2013). Further, in the helical conformation, all the side chains of amino acids are present on one side of the helix and the PTS2 helix is connected to the main protein via a flexible linker line region (Kunze et al., 2011; reviewed in Kunze, 2020). The PTS2 signal has been described by Kunze (2020) in great detail. PEX7 is a WD 40 repeat-containing protein, and it co-operates with specific co-receptors which are required for proper docking of the receptor cargo complex to the peroxisomal membrane (Lazarow, 2006). In most fungi, PEX7 interacts with PEX20 or with PEX18/PEX21, whereas in mammals and plants, it interacts with a splice variant of PEX5 called the PEX5 long form (Schliebs and Kunau, 2006). PEX7 forms a docking complex with PEX13 and PEX14 when delivering the PTS-containing protein into the peroxisome (Kim and Hettema, 2015). The docking complex is constituted by PEX13 and PEX14. PEX13 and PEX14 interact with each other and with the receptor cargo complex. The affinity of receptor cargo is more toward PEX14; therefore, PEX14 is assumed to be the entry site of the receptor cargo complex into the docking complex. PEX17 is a peripheral membrane protein, and it interacts strongly with PEX14 (Niederhoff et al., 2005; Kerssen et al., 2006).

In some species, the PTS2 signal is cleaved off after the protein is imported into the peroxisomal matrix (Tanaka et al., 2008). In the lumen of mammalians, plants, and *Yarrowia lipolytica* peroxisomes, PTS2 is proteolytically removed from the PTS2 proteins. The corresponding protease in mammals has recently been characterized as Tysand 1 (Kurochkin et al., 2007). In watermelon and *Arabidopsis thaliana*, the corresponding protein has been referred to as glyoxysomal processing protease (GPP) and DEG15 protease, respectively. *Arabidopsis thaliana* plant knockout for DEG15 shows a lack of PTS2 protein processing (Helm et al., 2007). The prediction analysis revealed the presence of a putative *Arabidopsis thaliana* DEG15 ortholog in *S. lycopersium* as well*.*

## PTS Independent Import: Piggybacking Import

Some of the peroxisomal proteins lack either PTS1 or PTS2. The mechanism by which they are imported to peroxisome was predicted to be piggybacking. This was first reported by Glover *et al.* (Glover et al., 1994), who reported that the N-terminal truncated version of thiolase (a PTS2 protein) was mislocalized to the cytosol, but the same was found to be localized in peroxisome if co-expressed with wild-type full-length thiolase. Isocitrate lyases from oilseed have been reported to be targeted to peroxisome via piggybacking (Lee et al., 1997). Kataya et al. (2015) reported the peroxisome targeting of protein phosphatase 2A holoenzyme via piggybacking. Similarly, McNew and Goodman (1994) reported that bacterial chloramphenicol acetyltransferase (CAT) subunits lacking PTS1 formed a complex with other CAT subunit-containing PTS1 in the cytosol and were imported into the peroxisome. Similar instances have also been reported in *Saccharomyces cerevisiae* (Yang et al., 2001; Gabay-Maskit et al., 2020).

## PTS Independent Import: Internal Peroxisomal Targeting Signal

Some of the peroxisomal matrix proteins have been reported to bear an I-PTS in addition to conventional PTS1 or PTS2 as well (van der Klei and Veenhuis, 2006). *Saccharomyces cerevisiae* carnitine acetyltransferase and *Hansenula polymorpha* alcohol dehydrogenase have been reported to have an additional I-PTS in addition to conventional PTS1. Pumpkin catalase also contains different versions of I-PTS, in the sense that it is imported to peroxisome exclusively by I-PTS, as it does not have any evident PTS1/PTS2 (Oshima et al., 2008). Till now, I-PTS is not understood in detail; however, I-PTS is not expected to be conserved across the species as PTS1 or PTS2 (Wolf et al., 2010). Further, Kempiński et al. (2020) have reported that acyl-CoA oxidase from *Saccharomyces cerevisiae*, which has neither a PTS1 nor a PTS2, is imported via a signal patch, rather than a linear sequence of amino acids.

### PTS Prediction Algorithms

Numerous proteins of eukaryotic cell organelles have been identified and functionally characterized using classical protein chemistry or forward and reverse genetics approaches. Similarly, in the case of peroxisomes, a significant number of additional proteins have been identified through the use of high-sensitivity proteome analyses in the past few years (Eubel et al., 2008; Reumann et al., 2009; Lingner et al., 2011; Quan et al., 2013). While performing subcellular proteomics, absolute purity cannot be achieved; rather, only high purity can be achieved, although it is challenging even to achieve high purity of the organelle or subcellular compartments. Due to its smaller size and fragile nature, it is challenging to isolate peroxisomes with high purity. Chloroplast and mitochondrial fractions are the common contaminants with peroxisomal fractions (Reumann et al., 2009). For peroxisome isolation, only a few model plant species are suitable, which also must be grown under standard conditions (Lingner et al., 2011). However, with the development of high-accuracy prediction tools, some of the experimental limitations can be overcome. These prediction tools might provide knowledge about plant peroxisomal matrix proteins (Reumann, 2011). Several prediction methods have been developed to predict and assemble PTS1 proteins from genomic sequences, but not many are developed for plants. A few prediction methods and algorithms such as PTS1 predictor (mendel.imp.ac.at/mendeljsp/sat/pts1/PTS1predictor.jsp), PeroxisomeDB (www.peroxisomedb.org), PeroxiP (www.bioinfo.se/PeroxiP/), AraPerox (), PredPlantPTS1 (ppp.gobics.de), and PPero (https://github.com/WangJueCUHK/PPero2.0) have been developed, primarily to predict peroxisome targeting protein from the available genomic sequences (Emanuelsson et al., 2003; Neuberger et al., 2003; Reumann, 2004; Hawkins and Bodén, 2005; Schlüter et al., 2009; Reumann, 2011; Reumann, 2012; Wang et al., 2017). However, all these prediction tools are restricted to PTS1 proteins only and primarily to canonical PTS1s, which form the bulk of PTS1 proteins. The primary constraints in the correct prediction of PTS1 are the dependency of PTS1 tripeptides on upstream target-enhancing residues, correct prediction of novel PTS1 tripeptides, and prediction of non-canonical PTS1 tripeptides (Lingner et al., 2011).

The accuracy of the prediction algorithm depends on the size, quality, and diversity of the example sequences used as a raw data set for model training. Unfortunately, the number of known PTS1 proteins is low for most model organisms, yielding a low set of data for the prediction algorithms. The low data set is the primary limiting factor in the development of robust prediction algorithms. Further, among the known PTS1s, the majority belong to the canonical type, which also makes the bulk of the raw data set, making it all the more difficult to predict non-canonical-type PTS1s (Reumann et al., 2007; Lingner et al., 2011; Reumann, 2011; Kunze, 2018).

### Peroxisomal Proteome in Plants

Plant peroxisomes unveil the significant extent of the functional convolution, flexibility, and specificity as it demonstrates the presence of innumerable peroxisomal pathways distinctive to the plant kingdom (Pan and Hu, 2018). Studying the peroxisomal proteome in plants is a necessary task to completely understand the functional aspects of the physiology of plants (Palma et al., 2009). The use of mass spectrometry (MS) for proteomic studies has remarkably extended our understanding of proteins and biochemical reactions in plant peroxisomes. Analysis of peroxisome proteome has been accomplished on a few selected species of plants, such as etiolated cotyledons (Fukao et al., 2002, 2003), green leaves (Reumann et al., 2007; Reumann et al., 2009), non-green suspension cell cultures (Eubel et al., 2008), etiolated seedlings (Quan et al., 2013) of *Arabidopsis thaliana*, cotyledons of etiolated *Glycine max* (Arai et al., 2008), leaves of *Spinacia oleracea* (Babujee et al., 2010), and fruits of *Capsicum annuum* (Palma et al., 2018). Further, Kaur and Hu (2011) predicted the peroxisome proteome of *Oryza sativa* based on its *Arabidopsis* counterpart. With the help of peroxisomal proteome analysis, an increased number of novel peroxisomal functions such as detoxification of methylglyoxal, biosynthesis of phylloquinone, catabolism of pseudouridine, biosynthesis of CoA, and expected regulatory proteins have been discovered (reviewed in Pan and Hu, 2018). Furthermore, the proteomic studies in plant peroxisome illustrate that the crucial protein content is preserved throughout the developmental process. Consequently, as formerly suggested (Pracharoenwattana and Smith, 2008), all plant peroxisomal classifications should be entitled as peroxisomes despite individual names such as leaf peroxisome, glyoxysome in seeds and germinating seedlings, and so on. Apart from all these important aspects, still, plant peroxisomes need to be explored much more at all higher levels as proteome analyses in many organs like roots and seeds have yet not been discovered and evenly the majority of monocots and dicots need to be explored. Still, peroxisomal membrane proteins are a challenging arena in proteomic studies.

## *Solanum lycopersicum* Peroxisome Proteome

*Solanum lycopersicum* (tomato) is a globally significant vegetable crop with an annual production of 180 million tons. The primary production areas are located near temperate climates. The production is largely affected by abiotic stresses such as drought, extreme temperature, high salinity, and cold in almost every stage of the life cycle of the tomato plant. There are certain wild species of tomato conferring considerable resistance to various abiotic stresses, but it is still a challenge to transfer those resistance traits to the commercially viable tomato species (Krishna et al., 2019). All the abiotic stress conditions have a common after effect of an increase in the concentration of reactive oxygen species (ROS), which causes oxidative damage to the cellular architecture. In plant cells, the ROS homeostasis is primarily coordinated by the chloroplast, mitochondria, and peroxisome (Mittler et al., 2004; Møller and Sweetlove, 2010; Habib et al., 2016; Zandalinas et al., 2020). Hence, in this paper, we intend to predict and analyze the peroxisome proteome of *S*. *lycopersicum*.

For the prediction of *S. lycopersicum* peroxisome proteome, the model plant *Arabidopsis thaliana* peroxisome proteome was used as a template (Reumann et al., 2007; Eubel et al., 2008; Kaur et al., 2011; Quan et al., 2013; Pan and Hu 2018). The *Arabidopsis* peroxisomal proteins were obtained from TAIR (Berardini et al., 2015), followed by protein BLAST at NCBI (Altschul et al., 1990). The closest orthologs obtained were taken as putative *S*. *lycopersicum* candidates (Supplementary Table S1). The candidate proteins were divided into three categories: putative PTS2, putative PTS1, and putative peroxisomal proteins without any obvious PTS. The putative *S*. *lycopersicum* orthologs were further predicted for peroxisomal localization using the Plant PTS1 Predictor database, and the results obtained are mentioned in column 16 (Supplementary Table S1, yes—meaning predicted to be peroxisomal, no—meaning predicted not to be peroxisomal). Depending upon the composition of C-terminus tripeptide (Reumann et al., 2016), the signals have been assigned a canonical or non-canonical nature in the case of both *Arabidopsis* (column 6, Supplementary Table S1) and *S*. *lycopersicum* (column 15, Supplementary Table S1). The canonical and non-canonical distinction in the case of *S*. *lycopersicum* entirely stems from the information available in the literature (Reumann and Weber, 2006) and remains predictive in nature. In the case of *Arabidopsis*, the candidate proteins have been reported to be present in the peroxisomal fraction, followed by identification using MS-based approaches, and/or demonstrated to be localized in peroxisomes via fluorescent fusion construct. However, the data obtained in the case of *S*. *lycopersicum* are predictive in nature.

### *Solanum lycopersicum* Probable PTS2 Proteins

*Arabidopsis thaliana* contains 19 PTS2 proteins (Reumann et al., 2009; Kaur and Hu 2011) in total belonging to various protein families. However, in the case of *S*. *lycopersicum*, the number was reduced to 14; nevertheless, the representative for each protein family was found (Supplementary Table S1). Acyl-CoA oxidase (ACX), an enzyme involved in fatty acid metabolism and jasmonic acid biosynthesis, was represented by three peroxisomal isoforms in *Arabidopsis thaliana*, namely, ACX3, ACX6, and ACX2, having PTS2 domains represented by RAx5HI, RAx5HI, and RIx5HL, respectively. The ACX protein family in *S*. *lycopersicum* was represented by two isoforms, having the putative PTS2 domains RTx5HL (in AtACX3 and AtACX6 ortholog) and RIx5HL (AtACX2 ortholog)*.* However, the presence of threonine in the second position in the PTS2 domain has yet to be demonstrated (Reumann and Chowdhary 2018); hence, this needs to be further experimentally validated. Another large *Arabidopsis thaliana* PTS2 protein family is thiolase (3-ketoacyl-CoA thiolase), represented by three peroxisomal isoforms. All the three *Arabidopsis thaliana* peroxisomal thiolases were represented by the same PTS2 domain, RQx5HL. In the case of *S*. *lycopersicum*, the peroxisomal thiolase family was represented by only one isoform; that is, acyl-CoA-acetyltransferase having the PTS2 domain was found to be RQx5HL. However, in the cases of both acyl-CoA oxidase and thiolase, the diversity and versatility of the PTS2 domain were found to be lesser in *S. lycopersicum* as compared to *Arabidopsis thaliana*.

Furthermore, NAD + malate dehydrogenase, citrate synthase, and long-chain acyl CoA synthase (LACS) family proteins were represented by two isoforms each in *Arabidopsis thaliana*. In the case of *S*. *lycopersicum*, NAD + malate dehydrogenase, citrate synthase, and long-chain acyl CoA synthase orthologs were represented by one isoform each. The PTS2 domains in the case of NAD + malate dehydrogenase and citrate synthase were found to be RIx5HL and RLx5HL, respectively, in the case of both *Arabidopsis thaliana* and *S. lycopersicum*. The long-chain acyl CoA synthase isoforms in the *Arabidopsis thaliana* contained RIx5HI and RLx5HI, while that of *S. lycopersicum* was found to be RLx5HL. Interestingly, out of the two LACS isoforms in *Arabidopsis thaliana*, one of them designated as LACS7 (At5g27600) also contained a canonical PTS1 represented by SKL> in addition to functional PTS2; however, the same in the case of *S. lycopersicum* was found to be an exclusively PTS2 protein.

Apart from these, the remaining PTS2 proteins in *Arabidopsis thaliana* having a probable ortholog in *S. lycopersicum* were naphthoate synthetase (NS), aspartate aminotransferase (AAT), and transthyretin-like protein (TLP). The PTS2 domains in the case of NS and AAT in *S. lycopersicum* were represented by RVx5HL and RLx5HL, respectively. The TLP in the case of *Arabidopsis thaliana* contained an internal PTS2 represented by RLx5HL, and the tomato ortholog also retained the peroxisomal signal as internal PTS2 represented by RVx5HL, demonstrating the conservation of peroxisomal targeting signals. Further, the *S. lycopersicum* orthologs of the alpha crystalline domain (ACD) and indigoidine synthase A (IndA) proteins were found to be uncharacterized proteins; however, both of them contained a canonical PTS2 represented by RVx5HL and RLx5HF, respectively. As per the KEGG (Kyoto Encyclopaedia of Genes and Genomics) metabolic database, the *S. lycopersicum* ortholog of indigoidine synthase A (XP_004250655.1) has yet to be assigned any probable metabolic function.

Interestingly, the histidine triad (HIT) family protein in *Arabidopsis thaliana* contained two PTS2-carrying isoforms (HIT2; AT5G48545 and HIT3; AT3G56490), and another isoform has no obvious PTS (HIT1; AT4G16566). The PTS2 domains in the case of HIT2 and HIT3 were represented by the canonical PTS2 signals RLx5HL and RVx5HF, respectively. The C-terminus tripeptide in the case of HIT1 was represented by AT*S*>. Serine at the -1 position yet remains to be proven as a functional PTS1 residue and threonine at -2 is a non-canonical PTS1 residue (Reumann et al., 2016), suggesting that ATS> may not be a functional PTS1 candidate and may get imported to the peroxisome matrix via some unknown mechanism. The sole reason for including this protein in the peroxisome proteome is that this protein was reported to be present in peroxisomal fractions in proteome analysis (Reumann et al., 2007; Eubel et al., 2008; Reumann et al., 2009; Guranowski et al., 2010). The *S. lycopersicum* HIT family ortholog was also represented by three isoforms; two of them (XP_004249357.1 and NP_001234539.2) had PTS2 domains represented by RLx5HF in both cases. The HIT1 ortholog of *S*. *lycopersicum* (XP_004244571.1) was predicted to contain a non-canonical PTS1 represented by SSM>. Considering the tripeptide residues present in the HIT protein family, the *S. lycopersicum* ortholog (SSM>) has a higher propensity to be targeted to peroxisome as compared to its *Arabidopsis thaliana* ortholog (AT*S*>) via PTS1. The tomato HIT PTS2 ortholog, NP_001234539.2, was also predicted to have two internal non-canonical PTS1 signals, SNI-13 and SSL-3; nevertheless, whether these internal PTSs are functional or not yet remains to be verified experimentally*.*

### Amino Acid Prevalence at Various PTS2 Positions

**FIGURE 1:**
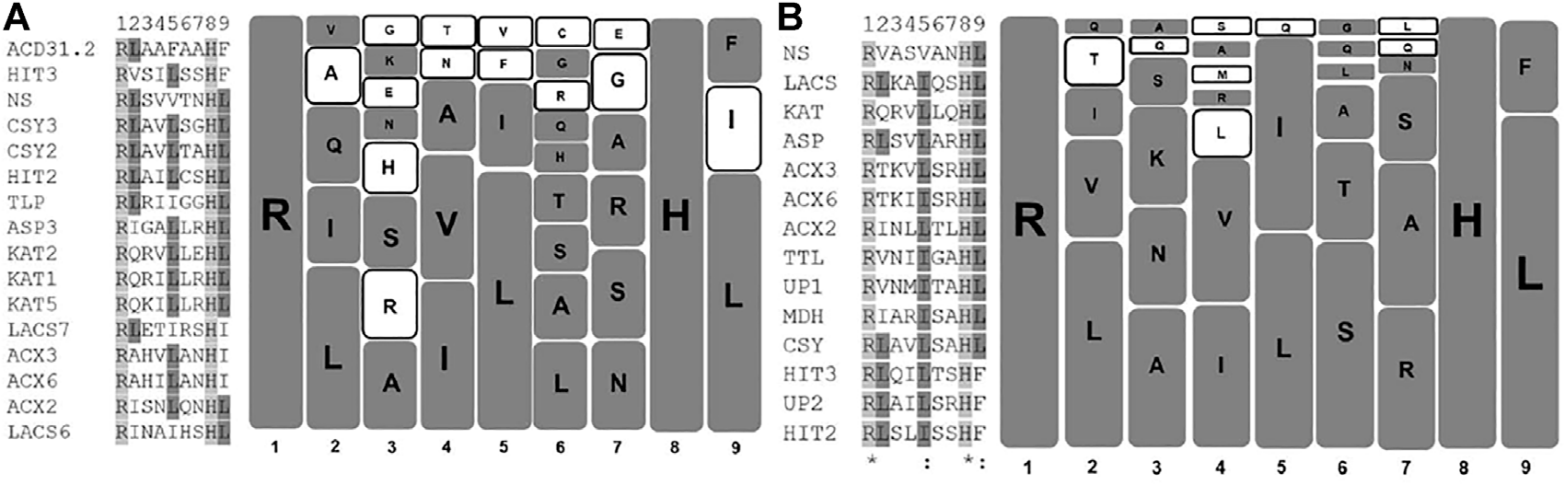
Graphical representation of PTS2 domains of *Arabidopsis thaliana* and *Solanum lycopersium*: Unique PTS2 sequences from *Arabidopsis thaliana* and *S. lycopersicum* were taken, and a multiple sequence alignment (MSA) was performed. In the case of *Arabidopsis thaliana* and *S. lycopersicum*, 16 and 14 unique PTS2 sequences were taken for MSA, respectively. The MSA was performed at pir.georgetown.edu using the clustalW program. The left half of the diagram reflects the MSA, while the right half represents the diagrammatic representation of the amino acids present at specific positions in *Arabidopsis thaliana* **(A)** and *S. lycopersicum* **(B)**. Each square represents one amino acid. The bigger the size of the squares, the higher the propensity of amino acid to remain present at that specific position. The empty square represents the amino acids that differ among the two species selected here for comparison. **(A)** AT, **(B)** SL.

In this section, we have developed a comparative amino acid prevalence pattern for PTS2 residues in *Arabidopsis thaliana* and *S. lycopersicum*. For this, 16 and 14 unique PTS2 domains, respectively, from *Arabidopsis thaliana* and *S. lycopersicum* were taken and multiple sequence alignment was performed (Figure 1). In the nonapeptide, the first and last amino acids were referred to as 1 and 9, respectively. We have determined the propensity of an amino acid residue to remain present at a specific position. A percentage value was assigned to a specific amino acid residue depending upon its presence in the number of PTS2 domain sequences in that specific position in the specific (*Arabidopsis thaliana*/*S. lycopersicum*) organism. The higher the propensity value, the higher the chance of the presence of that specific amino acid in that position. The first and eighth amino acid residues were always arginine and histidine, respectively, in the case of both *Arabidopsis thaliana* and *S. lycopersicum* in all the respective 16 and 14 PTS2 domain sequences, and hence, a propensity value of 100 was assigned. Both arginine and histidine are positively charged residues, suggesting that positively charged residues are preferred in the first and eighth positions. At the ninth position, leucine, isoleucine, and phenylalanine have been demonstrated to remain present to constitute a functional PTS2 domain. In the case of *Arabidopsis thaliana*, all three amino acid residues were found to be present with propensity values in the decreasing order of leucine (68.75), isoleucine (18.75), and phenylalanine (12.5). The same in the case of *S*. *lycopersicum* was restricted to leucine (78.5) and phenylalanine (21.4) only. Isoleucine has not yet been predicted to be present at the ninth position in *S. lycopersicum*. At the ninth position, the propensity of leucine being present was found to be the highest and the propensity of phenylalanine was found to be the lowest. Phenylalanine being a bulky aromatic amino acid exhibits the least flexibility; therefore, receptor interactions would be compromised, justifying its comparative lesser prevalence. In the second position, leucine has the highest propensity to remain present in the cases of both *Arabidopsis thaliana* and *S. lycopersicum*. In *Arabidopsis thaliana*, leucine is followed by isoleucine and glutamine, while in the case of *S. lycopersicum*, it was found to be valine. This suggests that in the second position, neutral amino acids are preferred. In the fourth position, isoleucine followed by valine is favored in the cases of both *Arabidopsis thaliana* and *S. lycopersicum*. Similarly, in the fifth position, leucine is followed by isoleucine in both organisms. The propensity of having leucine in the fifth position is relatively higher, ranging from 50 in *S. lycopersicum* to 68.75 in *Arabidopsis thaliana*. The third, sixth, and seventh positions showed relatively higher variations in the case of both the organisms (Table 1; Figure 1).

**TABLE 1:** Amino acid propensity at various positions of PTS2 in *Arabidopsis thaliana* and *Solanum lycopersicum*: PTS2 sequences from both the plant species were mined from the database and the specific amino acids present at various positions were listed.

Sl. No	Position		Amino acid		Number of times present		Propensity (in percentage)	
	SL	AT	SL	AT	SL	AT	SL	AT
1	1	**1**	R	**R**	14	**16**	100	**100**
2	2	**2**	L	**L**	6	**7**	42.8	**43.75**
3			V	**I**	3	**3**	21.4	**18.75**
4			I	**Q**	2	**3**	14.2	**18.75**
5			T	**A**	2	**2**	14.2	**12.5**
6			Q	**V**	1	**1**	7.14	**6.25**
7	3	**3**	A	**A**	4	**4**	28.5	**25**
8			N	**R**	3	**3**	21.4	**18.75**
9			K	**S**	3	**3**	21.4	**18.75**
10			S	**H**	2	**2**	14.2	**12.5**
11			Q/A	**N/E/K/G**	1	**1**	7.14	**6.25**
12	4	**4**	I	**I**	4	**6**	28.5	**37.5**
13			V	**V**	4	**5**	28.5	**31.25**
14			L	**A**	2	**3**	14.2	**18.75**
15			R/M/A/S	**N/T**	1	**1**	7.14	**6.25**
16	5	**5**	L	**L**	7	**11**	50	**68.75**
17			I	**I**	6	**3**	42.8	**18.75**
18			V	**F/V**	1	**1**	7.14	**6.25**
19	6	**6**	S	**L**	6	**4**	42.8	**25**
20			T	**A**	3	**3**	21.4	**18.75**
21			A	**S**	2	**2**	14.2	**12.5**
22			L/Q/G	**T**	1	**2**	7.14	**12.75**
23	—		—	**H/Q/R/G/C**		**1**	—	**6.25**
24	7	**7**	R	**N**	4	**4**	28.5	**25**
25			A	**S**	4	**4**	28.5	**25**
26			S	**R**	3	**3**	21.4	**18.75**
27			N/Q/L	**A**	1	**2**	7.14	**12.5**
28	—		—	**G**	—	**2**	—	**12.5**
29	—		—	**E**	—	**1**	—	**6.25**
30	8	**8**	H	**H**	14	**16**	100	**100**
31	9	**9**	L	**L**	11	**11**	78.5	**68.75**
32			F	**I**	3	**3**	21.4	**18.75**
33	—		—	**F**	—	**2**	—	**12.5**

The column “number of times present” describes the number of PTS2 sequences containing that specific amino acid at that specific position. Each amino acid at a specific position was assigned a percentage value referred to as propensity. If an amino acid was found to be present in all the PTS2 sequences (16 and 14 in *Arabidopsis thaliana* and *S. lycopersicum*, respectively) in a specific position, it was assigned 100% propensity value. AT, *Arabidopsis thaliana*; SL, *Solanum lycopersicum*. AT columns are written in bold.

## *Solanum lycopersicum* Putative PTS1 Proteins

As per the PTS1 proteome information of *Arabidopsis thaliana*, the proteins were categorized into various groups as per the (functions)/predicted functions: thioesterase, coumarate-CoA ligase, acyl-activating enzyme, aminotransferase, protease, catalase, phosphatases, kinases, uncharacterized proteins, reductases, dehydrogenase, and oxidases (Supplementary Table S1).

### Thioesterase Protein Family

The thioesterase family of proteins, involved in fatty acid metabolism and/or ubiquinone and other terpenoid-quinone biosynthesis pathways, was found to contain seven isoforms in *Arabidopsis thaliana*. Out of the seven members, six of them contained canonical PTS1, while one candidate protein, namely, the thioesterase family protein (At1g04290), contained non-canonical PTS1 represented by SNL> (”>” represents the end of the protein sequence, and “underlined residue” represents the non-canonical or low-abundance PTS1 residue). *S. lycopersicum* was predicted to contain orthologs for all the seven *Arabidopsis thaliana* thioesterase isoforms. The four *S. lycopersicum* orthologs for Acyl-CoA thioesterase (At1g01710), small thioesterase 3 (At3g61200), esterase/lipase/thioesterase family 1 (At5g11910), and Acyl-CoA thioesterase family protein (At4g00520) were found to be uncharacterized/hypothetical proteins represented by either non-canonical PTS1, PKL>, ASL>, and SRF> or a C-terminus tripeptide PML>, respectively. The three non-canonical tripeptides, PKL>, ASL> (Reumann et al., 2007), and SRF> (Lingner et al., 2011), have been demonstrated to be targeted to the peroxisome. The PML> tripeptide containing two non-canonical residues, proline and methionine, at -3 and -2 positions, respectively, have yet to be demonstrated to be a functional PTS1. However, previously, tripeptides with two non-canonical residues, SNV> and TNL>, present in *Zinnia elegans* acyl CoA oxidase isoform 4 and *Arabidopsis thaliana* glutathione reductase, respectively, were demonstrated to be peroxisomal (Kataya and Reumann 2010; Skoulding et al., 2015). The remaining three *Arabidopsis* protein family members for which defined orthologs were predicted contained canonical PTS1 represented by SKL> (>XP_004246762.1), SKM> (>XP_004241184.1), and AKL> (>XP_004232859.1). Interestingly, the only protein in the *Arabidopsis thaliana* thioesterase family, small thioesterase 4 (At1g04290), contained a non-canonical PTS1 (SNL>), and the putative counterpart for the same in *S. lycopersicum* was represented by acyl-coenzyme A thioesterase 13 (XP_004246762.1), which contained a canonical PTS1 (SKL>).

### Coumarate-CoA Ligase and the Acyl-Activating Enzyme Protein Family

*Arabidopsis thaliana* contained five and seven isoforms for Coumarate-CoA ligase and Acyl-activating enzyme, respectively. Both the enzyme families have been predicted to be involved in the terpenoid-quinone biosynthesis pathways. All the isoforms contained canonical PTS1 except one candidate protein, namely, acyl-activating enzyme 14 (At1G30520), which showed the presence of non-canonical PTS1 represented by SSL>. In the case of *S. lycopersicum*, coumarate-CoA ligase and acyl-activating enzyme putative orthologs were represented by four and six family members, respectively, and all the proteins contained canonical PTS1. The *S. lycopersicum* putative ortholog of *Arabidopsis thaliana* protein (acyl-activating enzyme 14; At1G30520) with non-canonical PTS1 was found to contain a canonical PTS1 represented by SRL>.

Interestingly, in *Arabidopsis thaliana*, in both the abovementioned protein families of thioesterase and acyl-activating enzyme, one candidate protein in each contained non-canonical PTS1 represented by SNL> and SSL> in thioesterase family protein (At1g04290) and acyl-activating enzyme 14 (At1g30520), respectively. However, the counterpart orthologs in *S. lycopersicum* contained canonical PTS1 in both cases represented by SKL> and SRL >, respectively. This exhibits a point mutation of asparagine → lysine and serine → arginine, respectively. This suggests that during the process of evolution, point mutation might have led to the conversion of non-canonical to canonical PTS1s. Also, the conversion of the non-canonical residue to the canonical residue is a constructive evolution, which makes the protein fully destined for peroxisome targeting. Also, as per the evolutionary lineage, Solanaceae (*S. lycopersicum*) lies above the Brassicaceae (*Arabidopsis thaliana*) (Goldberg, 1989), supporting the claim of constructive evolution.

### Aminotransferase and Protease Protein Family

Further, the aminotransferase and protease family in *Arabidopsis thaliana* consists of four and nine candidate proteins, respectively, while the putative orthologs in *S. lycopersicum* were represented by three and seven members, respectively. All the aminotransferases in both the plant species were represented by canonical PTS1s. The two *Arabidopsis thaliana* protease family isoforms, namely, ATP-dependent caseinolytic Clp protease/crotonase (AT2G30650 and AT2G30660), were also predicted to exhibit hydroxyisobutyryl CoA hydrolase (CHY) activity. In total, the *Arabidopsis thaliana* peroxisome proteome has been predicted to contain a total of three homologous proteins (CHY1-AT5G65940, CHYH1-AT2G30650, and CHYH2-AT2G30660) to show hydroxyisobutyryl CoA hydrolase (CHY) activity. In the case of *S. lycopersicum*, there lies only one ortholog which was predicted to exhibit hydroxyisobutyryl CoA hydrolase activity; however, the *S. lycopersicum* CHY1 ortholog has not been predicted to be involved in protease activity. The PTS1 domain in the case of both the plant species remains conserved and was represented by AKL>. *Arabidopsis thaliana* CHY1 protein (AT5G65940) has also been demonstrated to be involved in cold stress tolerance via the C-repeat binding factor (CBF)-mediated cold tolerance pathway. The *chy1* mutants have been shown to accumulate low levels of CBF3, thereby making the plant sensitive to cold and freezing stress. The mutant plants have also been shown to accumulate higher levels of reactive oxygen species (Dong et al., 2009). However, whether the CHY1 homologs, CHY1H1 or CHY1H2, could complement the loss of function of *chy1* in *Arabidopsis thaliana* is yet to be experimentally verified. On the other hand, the presence of only one isoform exhibiting hydroxyisobutyryl CoA hydrolase activity in *S*. *lycopersicum* suggests that a natural loss of function could be detrimental to the relative cold stress tolerance property of *S. lycopersicum*.

Among the protease family members, peroxisomal DEG protease, which is known to cleave the N-terminal of PTS2, once the protein has been imported into the peroxisomal matrix (Schuhmann and Adamska, 2012), was also reported to be present in *Arabidopsis thaliana*. *S. lycopersicum* also contained a putative ortholog for DEG protease; however, the experimental validation of *S*. *lycopersicum* protease remains to be done. The PTS1 domain in both the plant species in DEG protease was represented by canonical PTS1, SKL>. The presence of DEG protease and conservation of the PTS1 signal in both plant species suggests that this may be a universal feature across the plant groups.

### Catalase Family

*Arabidopsis thaliana* peroxisome proteome contained three catalase isoforms, while that of *S. lycopersicum* was restricted to two isoforms. However, in both cases, the internal PTS was found to be QKL-10>, suggesting that QKL-10 > could be a universal internal PTS in catalase proteins. Further, Oshima et al. (2008) have also reported that catalase from plant systems are imported in a PEX5-dependent manner; however, the catalase-PEX5 binding may be different from the typical PTS1 protein-PEX5 binding.

### Phosphatase and Kinase Family

Kataya et al. (2016) demonstrated the peroxisomal localization of putative purple acid phosphatase (PAP, At2g01880) 7 by fluorescent fusion construct. The PTS1 domain was represented by a non-canonical tripeptide AHL>. The putative *S. lycopersicum* ortholog of PAP7 was also represented by non-canonical PTS1, SNI>. Kataya et al. (2015), Kataya et al. (2016) also reported the peroxisomal localization of protein phosphatase 2A holoenzyme and protein phosphatase 2C family members POL like phosphatases PLL2 and PPL2 via canonical PTS1, SRL> and SRM >, respectively. Along with the phosphatases, kinases have also been reported to play an active role in the regulation of protein activities via the interplay of the activation/deactivation phenomenon. The *Arabidopsis thaliana* peroxisome proteome revealed the presence of five kinases, out of which only one of them, namely, glyoxysomal protein kinase (GPK1, At3g17420), was found to contain canonical PTS1 represented by AKI>. The putative *S. lycopersicum* ortholog of AtGPK1 was found to be a probable receptor-like protein kinase (XP_010313851) terminating with *H*QV>. Two of the *Arabidopsis thaliana* kinase family members, namely, PfkB-type carbohydrate kinase (At1g49350) and NADH kinase (At1g78590), were found to contain non-canonical PTS1 represented by SML> and SRY >, respectively. The putative *S*. *lycopersicum* ortholog of AtPfkB-type carbohydrate kinase was found to be an uncharacterized protein containing a canonical PTS1 represented by SKL>. The *Arabidopsis* protein has been shown to be a yeast homolog YeiC involved in pseudouridine catabolism (Preumont et al., 2008). The presence of canonical PTS1 in the *S*. *lycopersicum* ortholog suggests the conservation of pseudouridine catabolism in the peroxisome. The *S*. *lycopersicum* ortholog of NADH kinase was represented by the NADH kinase isoform terminating with V*VA*> (italicized amino acid represents that the residue has yet to be proven to be present in functional PTS1). Further, *Arabidopsis* also contained two more protein kinases, namely, nucleoside diphosphate kinase type 1 (NADPK1, At4g09320) and dephospho-CoA kinase (CoAE, At2g27490), terminating with *Y*E*T*> and *I*G*S* >, respectively (shown in the peroxisomal protein without any obvious PTS category in Supplementary Table S1). Both the C-terminus tripeptides are not likely to be PTS1s; however, their peroxisomal localization has been validated using a fluorescent fusion construct (Reumann et al., 2009). These proteins having no obvious PTS might be targeted to peroxisome via an alternate yet unexplained mechanism. These proteins are targeted via piggybacking mechanism, also cannot be ruled out. The *S*. *lycopersicum* orthologs in both the cases also contained no obvious PTSs. The localization of a considerable number of kinases and phosphatases in peroxisomes suggests the regulatory role of peroxisomes.

### Uncharacterized Proteins

*Arabidopsis thaliana* PTS1 proteome has been shown to contain five unknown/uncharacterized proteins (UPs). Three of the uncharacterized proteins with UP9 (At1g29120), UP7 (AT5G65400), and UP3 (AT2G31670) contained non-canonical PTS1 represented by ASL>, SLM>, and SSL >, respectively. The *S. lycopersicum* putative ortholog of UP9 was found to be a putative lipase, having a C-terminus tripeptide represented by PSL>. The *S. lycopersicum* putative ortholog of UP7 (AT5G65400) was predicted to be an esterase (AGAP003155) protein belonging to the serine hydrolase protein family. However, the C-terminus tripeptide of this protein was represented by STV>. In both the abovementioned *S*. *lycopersicum* proteins, two residues (proline and serine at -1 and -2 positions in the case of the UP9 ortholog, threonine and valine at -2 and -1 positions in UP7) within the C-terminus tripeptide were found to be non-canonical, suggesting that it has the least probability to be peroxisomal. These two *S*. *lycopersicum* orthologs yet remain to be experimentally verified. The *S. lycopersicum* putative ortholog of *Arabidopsis thaliana* UP3 (AT2G31670) was found to be a stress-responsive A/B barrel family protein having a non-canonical C-terminus tripeptide represented by ASL>. Furthermore, the remaining two uncharacterized/unknown proteins in *Arabidopsis thaliana* with accession numbers AT1G16730 and AT5G44250 were found to contain canonical PTS1s represented by SKL> and SRL >, respectively. The ortholog of *Arabidopsis thaliana* protein UP6 (At1g16730) was not found in *Solanum lycopersicum*, while that of UP5 (AT5G44250) was found to be an uncharacterized protein LOC101257658 (XP_004238167.1), containing a canonical PTS1. The putative functional annotations observed in the case of *S*. *lycopersicum* orthologs of *Arabidopsis thaliana* uncharacterized proteins could indicate probable functional relatedness.

### Novel PTS1 Candidate: Annexin

The exhaustive peroxisome proteome list which was used for comparative analysis in this paper was prepared based on Kaur and Hu. (2011) and compared with Eubel et al. (2008), Reumann et al. (2009), and Quan et al. (2013). However, none of the analyses reported the presence of annexin (At2g38760) in the peroxisomal proteome. In our independent bioinformatics analysis, we could observe *Arabidopsis* annexin to contain a canonical PTS1 represented by SKI>. The putative *S. lycopersicum* ortholog of *Arabidopsis* annexin was found to contain a non-canonical PTS1 represented by AKV>, which yet remains to be demonstrated to be peroxisomal via wet-lab experimentations. Annexin is a ubiquitous protein capable of calcium-dependent and calcium-independent binding to phospholipids (Boustead et al., 1989; De Carvalho-Niebel et al., 2002; Mortimer et al., 2008). Their involvement in abiotic stress tolerance via maintenance of calcium homeostasis and ROS in the cellular environment has also been proposed (Gerke et al., 2005).

## *Arabidopsis thaliana* versus *S. lycopersicum*: A Comparative Analogy of Peroxisome Matrix Proteome

**FIGURE 2:**
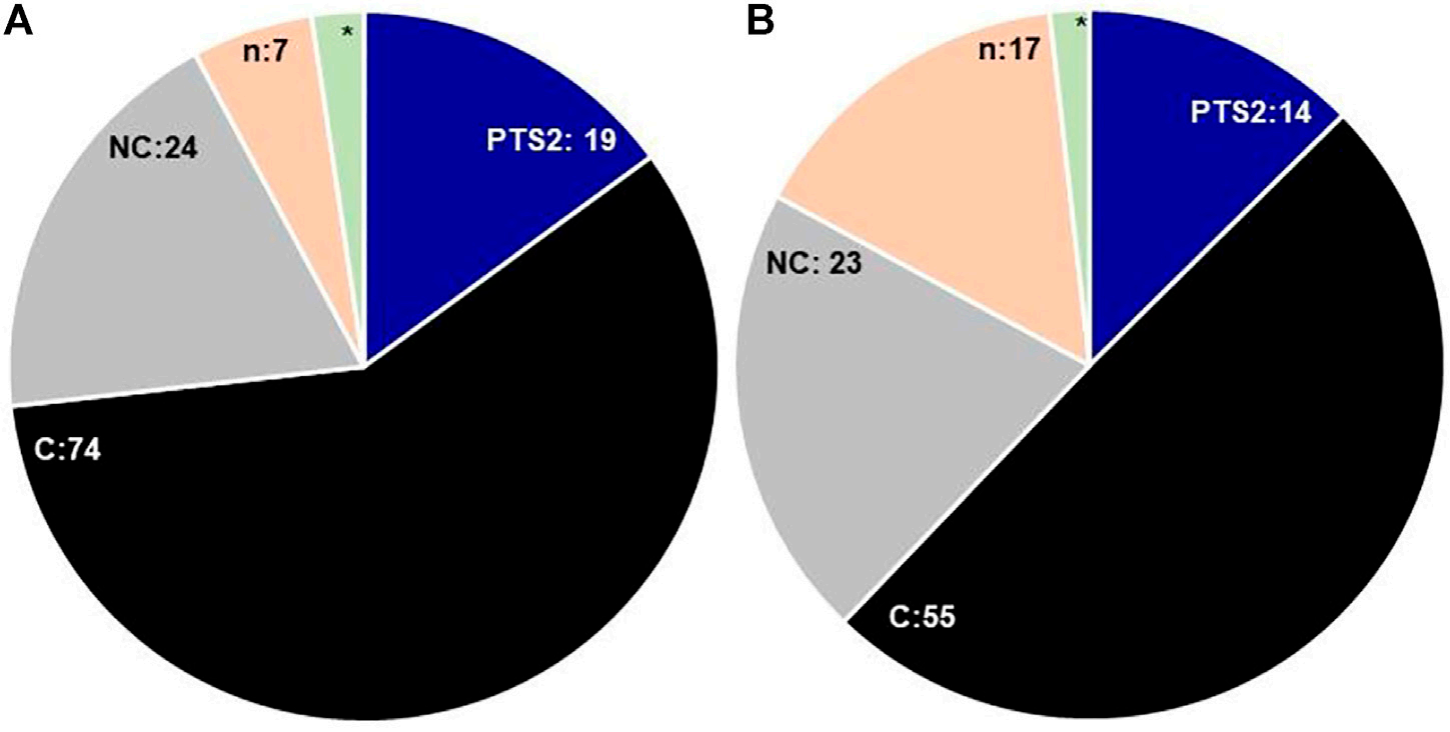
Comparative snapshot of *Arabidopsis thaliana* **(A)** and *Solanum lycopersicum* **(B)** peroxisomal proteome: the figure shows the comparative numbers of proteins/putative proteins present in each category; PTS1—peroxisomal targeting signal type 1 [C—canonical, NC—non-canonical], n—no obvious PTS, * - catalase containing internal PTS; PTS2: peroxisomal targeting signal type 2. The digits denote the respective numbers of proteins/putative proteins present in each category, * = 3 in *Arabidopsis thaliana*, 2 = *Solanum lycopersicum*.

In this paper, we cataloged 108 PTS1 proteins in *Arabidopsis thaliana*; 74 of these contained canonical PTS1, and 24 were found to have non-canonical PTS1 (Figure 2, showing a snapshot of peroxisomal matrix proteins in both the plant species). However, 10 candidate proteins were found with no predicted or “obvious” PTS, namely, catalase 1, catalase 2, catalase 3, cysteine proteinase (At4g36880; SSV>), glutathione reductase (At3g24170; TNL>), HIT1 (At4g16566; AT*S*>), cobalamin independent methionine synthase (ATMS1, At5g17920; SA*K*>), senescence-associated protein/B12D-related protein (B12D1, At3g48140; PTY>), nucleoside diphosphate kinase type 1 (NDPK1, At4g09320; *Y*E*T*>), and dephospho-CoA kinase (CoAE, At2g27490, IG*S*>). All the three isoforms of catalase possessed internal PTS represented by QKL-10>, which have been experimentally demonstrated to be peroxisomal in nature (Frederick and Newcomb, 1969; Frugoli et al., 1996; Zimmermann et al., 2006; Reumann et al., 2007; Eubel et al., 2008; Reumann et al., 2009). Further, Oshima et al. (2008) reported that plant catalases are imported to the peroxisome matrix in a PEX5-dependent manner, although the binding of catalase and PEX5 was found to be distinct from typical PTS1 proteins. The catalase orthologs in *S. lycopersicum* were represented by two isoforms and also predicted to contain internal PTS as QKL-10>, suggesting the conservation of internal PTS in the catalase protein family. *Arabidopsis thaliana* cysteine proteinase (SSV>) was reported in the proteomics study and was demonstrated to be peroxisomal using a fluorescent fusion construct (Quan et al., 2013). The *S*. *lycopersicum* ortholog was represented by cysteine proteinase RD21A-like with a C-terminus tripeptide SY*D*>, which yet needs to be demonstrated to be peroxisomal.

In the case of *Arabidopsis thaliana* glutathione reductase (GR), the C-terminus tripeptide was represented by TNL>, where only one residue, that is, leucine, present at the -1 position is canonical in nature, while the other two amino acid residues asparagine and threonine present at -2 and -3, respectively, are non-canonical in nature. The protein was reported to be peroxisomal in the proteomics studies (Eubel et al., 2008; Reumann et al., 2009). However, TNL> tripeptide was experimentally proven to be peroxisomal via fluorescent fusion construct (Kataya and Reumann, 2010). The *S. lycopersicum* GR ortholog also contained TNL> as a C-terminus tripeptide, suggesting that TNL> present in glutathione reductases could also be a universal peroxisomal targeting signal.

*Arabidopsis thaliana* HIT1 (At4g16566) and ATMS1 (At5g17920), where the C-terminus tripeptide is represented by AT*S*> and SA*K*>, respectively, in both the cases serine and lysine at the -1 position have yet to be demonstrated to be present in the functional PTS1 tripeptide motif. The amino acid residues at -2, threonine, and alanine are of non-canonical nature, while the -1 residues alanine and serine are of canonical nature. These two proteins have been reported to be present in peroxisomal fractions in proteome analysis (Fukao et al., 2002; Reumann et al., 2007; Eubel et al., 2008; Reumann et al., 2009; Guranowski et al., 2010). Further, HIT1 and ATMS1 were demonstrated to be localized in peroxisome via fluorescent fusion construct by Reumann et al. (2009) and Quan et al. (2013), respectively. Considering the composition of C-terminal tripeptide, it is very unlikely that these two proteins would be targeted via a PTS1-dependent mechanism; however, further experiments need to be done in this regard. The *S*. *lycopersicum* orthologs of AtHITA1 and ATMS1 were represented by bifunctional adenosine 5′-phosphosulfate phosphorylase/adenylyl sulfatase and 5-methyltetrahydropteroyltriglutamate-homocysteine methyltransferase, respectively. The former one was found to contain a non-canonical-type PST1 (SSM>), while the latter one did not have any obvious PTS1 and the C-terminus tripeptide was represented by SA*K*>, the same as its *Arabidopsis* counterpart. Conservation of the same C-terminus tripeptide (SA*K*>) suggests that the *S*. *lycopersicum* ortholog could also be targeted to peroxisome; however, it needs to be validated experimentally.

The *Arabidopsis thaliana* proteins B12D1, NDPK1, and CoAE were reported in proteomics studies and were also demonstrated to be peroxisomal via fluorescent fusion construct by Reumann et al. (2009). The *S*. *lycopersicum* orthologs for all three proteins did not contain any “obvious” PTS. The targeting of protein without any obvious peroxisome targeting signal could be due to some yet unknown or novel mechanism. However, the possibility of piggybacking is also not ruled out.

**FIGURE 3:**
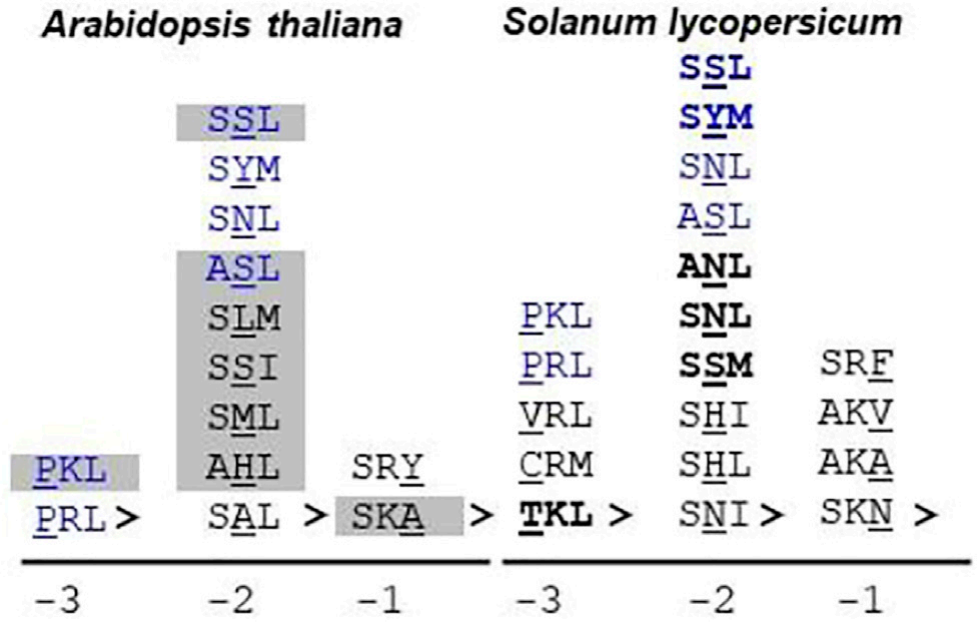
Non-canonical PTS1 sequences in *Arabidopsis thaliana* and *Solanum lycopersicum*: The non-canonical or low-abundance sequences are the ones that contain one non-canonical residue in the PTS1 tripeptide. The non-canonical residue could be present at -1, -2, or -3 positions. *S. lycopersicum* exhibits a greater diversity of non-canonical residues as compared to *Arabidopsis thaliana*. Blue text: non-canonical PTS1 present in both *Arabidopsis thaliana* and *S. lycopersicum*. Bold text: successfully predicted by the PTS1predictor algorithm (only *S*. *lycopersicum* proteins taken into account); gray highlighted tripeptide: demonstrated peroxisomal localization by fluorescent fusion construct; underlined residue: non-canonical residue; “>” indicates the end of the peptide sequence. The number at the bottom indicates the position of the non-canonical residue in the PTS1 tripeptide. Underlined residues are the non-canonical ones. For *S*. *lycopersicum*, no wet-lab data are available.

In *S. lycopersicum*, the putative orthologs for all the *Arabidopsis thaliana* proteins were found except At1g16730 (unknown protein 6); however, the number of isoforms was reduced, resulting in the overall reduction of putative PTS1 proteins to 97. The number of canonical PTS1s in *S. lycopersicum* was reduced to 55 as compared to 74 in *Arabidopsis thaliana*. However, the number of proteins possessing the non-canonical PTS1 in *Arabidopsis thaliana* and *S. lycopersicum* is comparable (24 and 23, respectively); nevertheless, the diversity of composition of the signal tripeptide is much higher in the case of *S*. *lycopersicum*, as shown in Figure 3.

**FIGURE 4:**
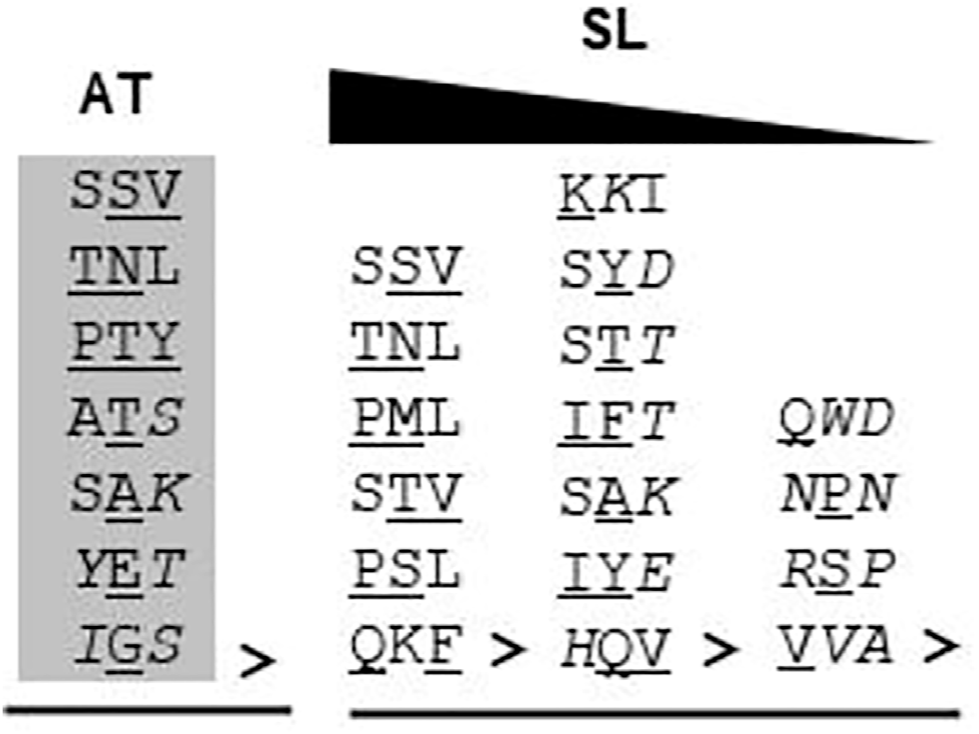
C-terminus tripeptide without any “obvious” PTS in *Arabidopsis thaliana* and *Solanum lycopersicum*: The C-terminus tripeptides which do not fall under either canonical or non-canonical categories were put under this. Gray highlighted tripeptide: demonstrated peroxisomal localization by fluorescent fusion construct; “>” indicates the end of the peptide sequence; underlined residue: non-canonical residue; italicized residue: amino acid not demonstrated to be functional PTS1; AT, *Arabidopsis thaliana*; SL, *S*. *lycopersicum*. For *S*. *lycopersicum*, no wet-lab data are available. The triangle at the top indicates the reducing propensity of tripeptides to be functional PTS1 from left to right.

Furthermore, in the case of *S. lycopersicum*, 19 proteins were predicted without any “obvious” PTS as against 10 in *Arabidopsis thaliana*. Whether these proteins are peroxisomal in nature or not needs to be experimentally validated. However, these 19 proteins have varied C-terminus tripeptide compositions – i. proteins having one canonical and two non-canonical PTS1 residues (PML>, TNL>, PSL>, STV>, QKF>, and SSV>); the probability of these being functional PTS1 cannot be ruled out as similar composition tripeptides have been reported to be functional PTS1 (TNL>, Kataya and Reumann, 2010, SSV>, Skoulding et al., 2015); ii. proteins having one amino acid residue not proven to be in the PTS1 tripeptide list (K*K*I>, SY*D*>, ST*T*>, IF*T*>, SA*K*>, IY*E*>, *H*QV>); and iii. proteins having more than one amino acid residue not proven to be in the PTS1 tripeptide list (Q*WD*>, *N*P*N*>, *R*S*P*>, and V*VA*>); the propensity of this category to be peroxisomal is the least and can only be verified experimentally, and also the chances of categories ii and iii to be peroxisomal (if at all) via PTS1 are least likely due to the composition of C-terminus tripeptide (Figure 4). All the three categories were also represented in *Arabidopsis thaliana* (SSV>, TNL>, PTY>, AT*S*>, SA*K*>, *Y*E*T*>, *I*G*S*>); however, all of them have been proven to be peroxisomal via fluorescent fusion construct, as explained earlier in this section.

### The Functionality of the Prediction Algorithm

As explained earlier, prediction algorithms have been developed only for PTS1 proteins, which are successful in predicting the canonical PTS1s due to their high abundance nature. The reliable prediction of PTS2 proteins and peroxisomal proteins with any obvious PTS still remains elusive (Kunze 2018). In our study, we compared three prediction algorithms, and Plant PTS1Predictor (Reumann et al., 2012) was found to be performing the best; hence, in the further analysis, we would be considering only the results of Plant PTS1Predictor. In the case of *S. lycopersicum*, only one PTS2 candidate, namely, the *Arabidopsis thaliana* ortholog of the alpha crystalline domain, was predicted to be peroxisomal. It is unusual for a PTS1 prediction algorithm to predict PTS2 protein; the possible reason could be that C-terminus tripeptide in this protein is represented by PKL>, which is very closely related to canonical PTS1 such as SKL> and PKL> and was also demonstrated to be localized in peroxisome via fluorescent fusion construct (Eubel et al., 2008). In the case of the PTS1 proteins, all the canonical PTS1 were successfully predicted to be peroxisomal by the algorithm. The algorithm was able to predict some of the non-canonical PTS1 correctly, such as SYM>, SSL>, ANL>, SSM>, TKL>, and SNL>. The result obtained from the prediction algorithm, and previous proteomic and localization analysis revealed that the presence of a non-canonical residue at the -2 position is preferable as compared to the same at either -1 or -3 positions. This could be due to the binding chemistry between the PTS1 cargo and the PEX5 receptor. Structurally, PEX5 is a bipartite protein having a disordered N-terminal domain (NTD) which harbors seven WxxxF/Y motifs and one LVAEF motif responsible for recognition of the PEX14 receptor present at the peroxisomal membrane (Carvalho et al., 2006; Neuhaus et al., 2014). The other half of protein is composed of seven tetratricopeptide repeat (TPRs), followed by a helical structure, and is responsible for the binding of the PTS1 cargo. The PTS1 binding pocket is constituted of two TPR triplets, TPR1-3 and TPR5-7, connected by TPR4. In PTS1 cargo binding, four asparagine amino acids present in TPRs play a very crucial role in terms of hydrogen bond formation with PTS1 residues. The PTS1 binding pocket favors small side-chain amino acids at -3 positions, while at -2 positions, basic side chains are favored (Gatto et al., 2000; Stanley et al., 2006). The PTS1 binding pocket and PTS1 cargo act as an induced-fit system, leading to a firm binding (Fodor et al., 2012). The arrangement of the PTS1 cargo binding pocket allows some structural flexibility at -2 positions. As depicted in Figure 3, *S. lycopersicum* exhibits greater diversity in the composition of non-canonical PTS1 tripeptides as compared to *Arabidopsis thaliana*. In both the plant species, six different non-canonical PTS1s represented by SSL>, SYM>, SNL>, ASL>, PKL>, and PRL> were found to be common. Apart from these, 7 and 13 unique non-canonical PTS1s were found in *Arabidopsis thaliana* and *S. lycopersicum*, respectively. In *Arabidopsis thaliana*, seven different non-canonical residues were found at the -2 position as against two different residues each at the -3 and -1 positions, respectively. Similarly, in the case of *S. lycopersicum*, four different non-canonical residues were observed at -2, as against three and two different residues at -3 and -1, respectively. Among all the non-canonical residues, SSL> was found to be represented in seven different proteins, including both *Arabidopsis thaliana* and *S. lycopersicum*. Apart from being present in SSL>, serine was found to be present at the -2 position in other PTS1 tripeptide compositions as well, such as ASL>, SSI>, and SSM>. In total, in our study, serine was found to be present in 14 different C-terminus tripeptide sequences including both *Arabidopsis thaliana* and *S. lycopersicum*. Further, SSL> has also been verified as a peroxisomal tripeptide using fluorescent fusion construct. Hence, we suggest that serine at the -2 position could be considered another canonical residue.

## Conclusion

Peroxisome being a very fragile organelle poses a lot of challenges in experimentations. However, in the past 2 decades, mass spectrometry-based applications have led to considerable advancement toward the knowledge of peroxisome proteome. However, still, a lot remains to be deciphered. Due to the lack of sufficient raw data, it has not been possible to develop robust algorithms for predictions. Here, we have predicted a detailed account of the peroxisome proteome of the economically important vegetable crop *S. lycopersicum* derived on the basis of the model plant *Arabidopsis thaliana*. However, the data obtained in the case of *S. lycopersicum* are predictive in nature and in the need of experimental verification. The putative PTS2 category showed a significant reduction in number in the cases of *S. lycopersicum* as compared to *Arabidopsis thaliana*; all the protein categories were represented, but the number of isoforms was found to be reduced. This suggests that during evolution, the redundancy may have been reduced. Further, in the case of *Arabidopsis thaliana*, all the PTS2 signals were canonical in nature, while in the case of the *S. lycopersicum* ACX3 and ACX6 orthologs, the signal was represented by RTx5HL. Threonine at the second position in the type 2 signal has yet to be experimentally established. The considerably lower number of PTS2 proteins and complexity in the signal (a nonapeptide) are the primary reasons for not having any successful prediction algorithm for PTS2 proteins.

The number of PTS1 proteins remained comparatively similar. In the case of *Arabidopsis thaliana*, 68% of proteins exhibited a canonical type 1 signal, while this percentage was reduced to 56 in the case of *S. lycopersicum*. The number of non-canonical type 1 signals was found to be almost similar in both cases. The major difference was observed in the number of proteins having no “obvious” PTS. This number was found to be 7 in the case of *Arabidopsis thaliana*, while in the case of *S. lycopersicum*, it was staggeringly high at 17. However, this could also be due to the lack of experimental validations in *S. lycopersicum*, upon which newer PTS1 tripeptides could be identified, which would further enrich the peroxisome signal database.

As per an *in silico* analysis conducted on the *Arabidopsis thaliana* genome, it has been predicted that the number of peroxisomal proteins could be higher than 400 (Lingner et al., 2011). However, despite substantial efforts, the peroxisome proteome is still far from near completion even in the case of the model plant *Arabidopsis thaliana*. However, the *Arabidopsis thaliana* data could be used as a baseline for building the peroxisome proteome resource for other crops of economic importance. The data generated here make a solid foundation for the peroxisome proteome research in the vegetable crop *S*. *lycopersicum*. This would also help in enriching the raw data for the preparation of prediction algorithms and ultimately result in better prediction algorithms.
