# Supplementary material for: Translating the Arabidopsis thaliana Peroxisome Proteome Insights to Solanum lycopersicum: Consensus Versus Diversity
Source: Front Cell Dev Biol. 2022 Jul 13;10:909604. doi: 10.3389/fcell.2022.909604 (PMC9328179; doi:10.3389/fcell.2022.909604)
Supplement: Supplementary file 1 [file Table1.DOCX]

# Translating the *Arabidopsis thaliana* peroxisome proteome insights to *Solanum lycopersicum*: consensus vs diversity

*Sabiha Tarafdar* *and Gopal Chowdhary^*^*

*Plant Molecular Biology laboratory, School of Biotechnology, KIIT, Bhubaneswar - 751024, Odisha, India.*

**Corresponding author: E-mail address:* [*gkchowdhary@kiitbiotech.ac.in*](mailto:gkchowdhary@kiitbiotech.ac.in)

**TABLE 1:** Peroxisomal matrix protein in *Arabidopsis* *thaliana* and *Solanum* *lycopersicum*: Listed are *Arabidopsis thaliana* peroxisomal matrix proteins derived from various literature resources (Eubel et al., 2008; Reumann et al., 2009; Kaur and Hu 2011; Quan et al., 2013). The AT proteins were used as a query to obtain putative SL orthologs. AT: *Arabidopsis thaliana*; SL: *Solanum* *lycopersicum*; C: canonical; NC: non-canonical; n: no obvious PTS; 1: Eubel et al., 2008; 2: Reumann et al., 2009; 3: Quan et al., 2013; 4: reviewed in Kaur and Hu 2011; 5: results from Plant PTS1Predictor database; P: proteomics study; L: subcellular localization study using fluorescent fusion construct; F: full-length protein used for subcellular localization; D: PTS domain used for fluorescent localization; Y: present in the specific analysis; x: absent in the specific analysis; i: internal PTS1; grey highlight: same SL orthologs.

|  | ***Arabidopsis thaliana*** | | | | | | | | | | ***Solanum lycopersicum*** | | | | |
| --- | --- | --- | --- | --- | --- | --- | --- | --- | --- | --- | --- | --- | --- | --- | --- |
|  | **Gene Name** | **At Locus** | **Protein Annotation** | **Nona/Tri-peptide** | **C/NC** | **L** | **1P** | **2P** | **3P** | **4** | **Acc. No.** | **Protein Annotation** | **Nona/Tri-peptide** | **C/NC** | 5 |
| **PTS2 category** | | | | | | | | | | | **Putative PTS2 category** | | | | |
|  | ACX3 | At1g06290 | Acyl-CoA oxidase 3 | RA(HVLAN)HI |  |  | Y | Y | Y | Y | XP_004248184.1 | Acyl-coenzyme A oxidase 3, peroxisomal-like isoform X1 | RT(KVLSR)HL |  | No |
|  | ACX6 | At1g06310 | Acy-CoA oxidase 6 | RA(HILAN)HI |  |  | x | x | x | Y | XP_004248185.1 | Acyl-coenzyme A oxidase 3, peroxisomal-like isoform X2 | RT(KIISR)HL |  | No |
|  | ACX2 | At5g65110 | Acyl-CoA oxidase 2 | RI(QRLSL)HL |  |  | x | Y | Y | Y | XP_004237411.1 | Acyl-coenzyme A oxidase 2, peroxisomal | RI(NLLTL)HL |  | No |
|  | KAT1 | At1g04710 | 3-Ketoacyl-CoA thiolase 1 | RQ(RILLR)HL |  |  | Y | Y | Y | Y | NP_001352962.1 | Acetyl-CoA C-acetyltranferase 5 | RQ(RVLLQ)HL |  | No |
|  | KAT2 | At2g33150 | 3-Ketoacyl-CoA thiolase 2 | RQ(RVLLE)HL |  |  | Y | Y | Y | Y | NP_001352962.1 | Acetyl-CoA C-acetyltranferase 5 | RQ(RVLLQ)HL |  | No |
|  | KAT5 | At5g48880 | 3-Ketoacyl-CoA thiolase 5 | RQ(KILLR)HL |  |  | x | Y | x | Y | NP_001352962.1 | Acetyl-CoA C-acetyltranferase 5 | RQ(RVLLQ)HL |  | No |
|  | MDH1 | At2g22780 | NAD+ malate dehydrogenase 1 | RI(ARISA)HL |  |  | Y | Y | Y | Y | NP_001234005.1 | Glyoxisomal malate dehydrogenase | RI(ARISA)HL |  | No |
|  | MDH2 | At5g09660 | NAD+ malate dehydrogenase 2 | RI(ARISA)HL |  |  | Y | Y | Y | Y | NP_001234005.1 | Glyoxisomal malate dehydrogenase | RI(ARISA)HL |  | No |
|  | CSY3 | At2g42790 | Citrate synthase 3 | RL(AVLSG)HL |  |  | Y | Y | Y | Y | XP_004251813.1 | Citrate synthase, glyoxysomal | RL(AVLSA)HL |  | No |
|  | CSY2 | At3g58750 | Citrate synthase 2 | RL(AVLTA)HL |  |  | Y | Y | Y | Y | XP_004251813.1 | Citrate synthase, glyoxysomal | RL(AVLSA)HL |  | No |
|  | LACS6 | At3g05970 | Long chain acyl-CoA synthetase 6 | RI(NAIHS)HL |  |  | Y | Y | Y | Y | XP_004230201.1 | [Long chain acyl-CoA synthetase 6, peroxisomal](https://www.ncbi.nlm.nih.gov/protein/XP_004230201.1/) | RL(KAIQS)HL |  | No |
|  | LACS7 | At5g27600 | Long chain acyl-CoA synthetase 7 | RL(ETIRS)HI |  |  | Y | Y | Y | Y | XP_004230201.1 | [Long chain acyl-CoA synthetase 6, peroxisomal](https://www.ncbi.nlm.nih.gov/protein/XP_004230201.1/) | RL(KAIQS)HL |  | No |
|  | HIT3 | At3g56490 | Histidine triad family protein 3 | RV(SILSS)HF |  | 2F | x | Y | x | Y | XP_004249357.1 | 14 kDa zinc-binding protein | RL(QILTS)HF |  | No |
|  | HIT2 | At5g48545 | Histidine triad family protein 2 | RL(AILCS)HL |  | 2F | x | Y | x | Y | NP_001234539.2 | Histidine triad family protein | RL(SLISS)HF |  | No |
|  | NS | At1g60550 | Naphthoate syntatase | RL(SVVTN)HL |  |  | x | Y | Y | Y | XP_004238650.1 | 1,4-dihydroxy-2-naphthoyl-CoA synthase, peroxisomal | RV(ASVAN)HL |  | No |
|  | ASP3 | At5g11520 | Aspartate aminotransferase | RI(GALLR)HL |  | 1D | Y | Y | Y | Y | XP_004245266.1 | Aspartate aminotransferase, cytoplasmic | RL(SVLAR)HL |  | No |
|  | TLP | At5g58220 | Transthyretin-like protein | RL(RIIGG)HL |  |  | Y | Y | x | Y | XP_010323150.1 | Uric acid degradation bifunctional protein TTL isoform X2 | RV(NIIGA)HL |  | No |
|  | ACD31.2 | At1g06460 | Alpha Crystalline domain protein | RL(AAFAA)HF |  |  | x | Y | x | Y | XP_004243244.1 | Uncharacterized protein LOC101259555 isoform X2 | RV(NMITA)HL |  | Yes |
|  | IndA | At1g50510 | Indigoidine synthase A | RI(SNLQN)HL |  | 2F | Y | Y | Y | Y | XP_004250655.1 | Uncharacterized protein LOC101255656 isoform X3 | RL(AILSR)HF |  | No |
| **PTS1 category** | | | | | | | | | | | **Putative PTS1 category** | | | | |
| Thioesterase protein family | ACH2 | At1g01710 | Acyl-CoA thioesterase | SKL> | C |  | Y | Y | Y | Y | XP_004229898.1 | Uncharacterized protein LOC101251817 | PKL> | **NC** | No |
|  | ACH2 | At4g00520 | Acyl-CoA thioesterase family protein | AKL> | C |  | Y | x | x | Y | XP_004229899.1 | Uncharacterized protein LOC101252120 | PML> | n | No |
|  | sT4 | At1g04290 | Thioesterase family protein | SNL> | **NC** |  | Y | Y | x | Y | XP_004246762.1 | Acyl-coenzyme A thioesterase 13 | SKL> | C | Yes |
|  | st1 | At1g48320 | Small thioesterase 1 | AKL> | C | 2F | x | Y | Y | Y | XP_004232859.1 | 1,4-dihydroxy-2-naphthoyl-CoA thioesterase 1 | AKL> | C | Yes |
|  | st5 | At2g29590 | Small thioesterase 5 | SKL> | C | 2F | x | Y | x | Y | XP_004241184.1 | Acyl-coenzyme A thioesterase 13-like | SKM> | C | Yes |
|  | st3 | At3g61200 | Small thioesterase 3 | SKL> | C | 2F | Y | Y | Y | Y | XP_004229829.1 | Uncharacterized protein LOC101257302 | ASL> | **NC** | No |
|  | ELT1 | At5g11910 | Esterase/lipase/thioesterase family 1 | SRI> | C | 2F | x | Y | x | Y | XP_010325033.1 | Uncharacterized protein LOC101257586 | SRF> | **NC** | No |
| Coumarate CoA ligase family | 4Cl3 | At1g20480 | 4-Coumarate:CoA ligase 3 | SKL> | C |  | Y | x | x | Y | XP_004253129.1 | 4-coumarate--CoA ligase-like 5 | SKL> | C | Yes |
|  | OPCL1 | At1g20510 | OPC-8:0 ligase 1 (4-Coumarate:CoA ligase activity) | SKL> | C |  | Y | Y | x | Y | XP_004253129.1 | 4-coumarate--CoA ligase-like 5 | SKL> | C | Yes |
|  | 4Cl5 | At4g19010 | 4-Coumarate:CoA ligase 5 | SRL> | C |  | x | x | x | Y | XP_004245937.1 | 4-coumarate--CoA ligase-like 6 | SKL> | C | Yes |
|  | 4CL2 | At5g63380 | **4-Coumarate:CoA ligase 2** | SKL> | C |  | x | Y | x | Y | XP_004236342.1 | 4-coumarate--CoA ligase-like 9 | ARL> | C | Yes |
|  | 4CL1 | At4g05160 | 4-Coumarate:CoA ligase 1 | SKM> | C | 2F | Y | Y | Y | Y | XP_004251107.1 | 4-coumarate--CoA ligase-like 7 | SKI> | C | Yes |
| Acyl-Activating Enzyme Protein Family | AAE1 | At1g20560 | Acyl-activating enzyme 1 | SKL> | C |  | Y | Y | x | Y | XP_004240530.1 | Probable acyl-activating enzyme 1, peroxisomal | SKL> | C | No |
|  | AAE18 | At1g55320 | Acyl-activating enzyme 18 | SRI> | C |  | x | x | x | Y | XP_004252244.1 | Probable acyl-activating enzyme 17, peroxisomal | SRL> | C | Yes |
|  | AAE17 | At5g23050 | Acyl-activating enzyme 17 | SKL> | C |  | Y | Y | x | Y | XP_004252244.1 | Probable acyl-activating enzyme 17, peroxisomal | SRL> | C | Yes |
|  | AAE5 | At5g16370 | Acyl-activating enzyme 5 | SRM> | C | 3F | Y | Y | Y | Y | XP_004231632.1 | Probable acyl-activating enzyme 6 | SRM> | C | Yes |
|  | AAE14 | At1g30520 | Acyl-activating enzyme 14 | SSL> | **NC** |  | x | x | x | Y | XP_004233163.1 | 2-succinylbenzoate--CoA ligase, chloroplastic/peroxisomal | SRL> | C | Yes |
|  | AAE12 | At1g65890 | Acyl-activating enzyme 12 | SRL> | C |  | x | x | x | Y | XP_004234491.1 | Butyrate--CoA ligase AAE11, peroxisomal-like | SRL> | C | No |
|  | AAE7 | At3g16910 | Acyl-activating enzyme 7 | SRL> | C |  | Y | Y | x | Y | XP_004252919.1 | Acetate/butyrate--CoA ligase AAE7, peroxisomal | SRL> | C | Yes |
| Catalase family | CAT3 | At1g20620 | Catalase 3 | QKL-10> | i |  | Y | Y | Y | Y | XP_004238430.1 | Catalase isozyme 3 | QKL-10> | i | No |
|  | CAT1 | At1g20630 | Catalase 1 | QKL-10> | i |  | Y | Y | Y | Y | XP_004238430.1 | Catalase isozyme 3 | QKL-10> | i | No |
|  | CAT2 | At4g35090 | Catalase 2 | QKL-10> | i |  | Y | Y | Y | Y | NP_001234186.2 | Catalase isozyme 2 | QKL-10> | i | No |
| Amino Transferase | GGT1 | At1g23310 | Glutamate-glyoxylate aminotranferase 1 | SKM> | C |  | Y | Y | Y | Y | XP_004239121.1 | Glutamate--glyoxylate aminotransferase 2 | SRM> | C | Yes |
|  | GGT2 | At1g70580 | Glutamate-glyoxylate aminotransferase 2 | SRM> | C |  | Y | Y | Y | Y | XP_004239121.1 | Glutamate--glyoxylate aminotransferase 2 | SRM> | C | Yes |
|  | SGAT1 | At2g13360 | Serine-glyoxylate aminotransferase | SRI> | C |  | Y | Y | x | Y | NP_001265946.1 | Hop-interacting protein THI032 | SRI> | C | Yes |
|  | AGT2 | At4g39660 | Alanine:glyoxylate aminotransferase 2 | SRL> | C |  | x | x | x | Y | XP_004237433.1 | Alanine--glyoxylate aminotransferase 2 homolog 1, mitochondrial isoform X1 | SKL> | C | Yes |
| Protease Protein Family | CHYH1 | At2g30650 | ATP-dependent caseinolytic Clp protease | AKL> | C |  | x | x | x | Y | XP_004229511.1 | 3-hydroxyisobutyryl-CoA hydrolase 1 | AKL> | C | Yes |
|  | CHYH2 | At2g30660 | ATP-dependent caseinolytic Clp protease | AKL> | C |  | x | x | x | Y | XP_004229511.1 | 3-hydroxyisobutyryl-CoA hydrolase 1 | AKL> | C | Yes |
|  | CHY1 | At5g65940 | 3-Hydroxyisobutyryl CoA hydrolase | AKL> | C |  | Y | Y | Y | Y | XP_004229511.1 | 3-hydroxyisobutyryl-CoA hydrolase 1 | AKL> | C | Yes |
|  | DEG15 | At1g28320 | Deg/HtrA protease | SKL> | C |  | Y | x | x | Y | NP_001307361.1 | Peroxisomal leader peptide-processing protease | SKL> | C | No |
|  | CP | At3g57810 | Cysteine protease | SKL> | C |  | x | x | x | Y | XP_019071440.1 | OTU domain-containing protein At3g57810 isoform X1 | SKL> | C | Yes |
|  | LON2 | At5g47040 | Lon protease homolog 2 | SKL> | C | 2F | x | Y | Y | Y | XP_010325534.1 | Lon protease homolog 2, peroxisomal isoform X1 | SKL> | C | Yes |
|  | PM16 | At2g41790 | Peptidase family M16 | PKL> | **NC** |  | Y | Y |  | Y | CAC67408.1 | Insulin degrading enzyme | VRL> | **NC** | No |
|  | SCPL20 | At4g12910 | Serine carboxypeptidase-like 20 | SKI> | C | 3F | x | x | Y |  | XP_004242437.1 | Serine carboxypeptidase-like 20 | KKI> | **n** | Yes |
|  | RDL | At4g36880 | Cysteine proteinase | SSV> | n | 3F | x | x | Y |  | XP_004230752.1 | Cysteine proteinase RD21A-like | SYD> | **n** | No |
| Reductases | SDRc | At3g01980 | Short chain dehydrogenase/reductase c | SYM> | **NC** |  | x | Y | Y | Y | XP_004243555.1 | Uncharacterized protein LOC101255117 | SYM> | **NC** | Yes |
|  | SDRb | At3g12800 | Short-chain dehyrogenase/reductase b | SKL> | C | 1D | Y | Y | Y | Y | XP_004243970.1 | Peroxisomal 2,4-dienoyl-CoA reductase | SKL> | C | Yes |
|  | SDR | At3g55290 | Short chain dehydrogenase/reductase c | SSL> | **NC** | 3F | Y | Y | Y | Y | XP_004249318.1 | Uncharacterized protein LOC101247479 | SSL> | **NC** | Yes |
|  | NQR | At1g49670 | NADH:quinone reductase | SRL> | C | 1D | Y | Y | Y | Y | XP_004230783.1 | Probable quinone oxidoreductase | AKL> | C | Yes |
|  | HPR | At1g68010 | Hydroxypyruvate reductase 1 | SKL> | C |  | Y | Y | Y | Y | XP_004231047.1 | Glycerate dehydrogenase | SKL> | C | Yes |
|  | OPR3 | At2g06050 | 12-Oxophytodienoate reductase 3 | SRL> | C |  | Y | Y | Y | Y | NP_001233873.1 | 12-oxophytodienoate reductase 3 | SRL> | C | Yes |
|  | MDAR1 | At3g52880 | Monodehydroascorbate reductase 1 | AKI> | C |  | Y | Y | x | Y | NP_001318117.1 | Monodehydroascorbate reductase | SKI> | C | Yes |
|  | GR1 | At3g24170 | Glutathione reductase 1 | TNL> | **n** |  | Y | Y | x | Y | NP_001308322.1 | Glutathione reductase | TNL> | **n** | Yes |
| Dehydrogenases | NDA2 | At2g29990 | NADPH dehydrogenase A2 | SRI> | C |  | x | x | x | Y | XP_004240404.1 | Internal alternative NAD(P)H-ubiquinone oxidoreductase A1, mitochondrial | SRI> | C | Yes |
|  | NDA1 | At1g07180 | NADPH dehydrogenase A1 | SRI> | C |  | x | x | x | Y | XP_004240404.1 | Internal alternative NAD(P)H-ubiquinone oxidoreductase A1, mitochondrial | SRI> | C | Yes |
|  | NDB1 | At4g28220 | NADPH dehyrogenase B1 | SRI> | C |  | x | x | x | Y | XP_004248145.1 | External alternative NAD(P)H-ubiquinone oxidoreductase B1, mitochondrial | SRI> | C | Yes |
|  | ICDH | At1g54340 | NADP-dependent isocitrate dehydrogenase | SRL> | C | 1D | Y | Y | Y | Y | XP_004228607.1 | Isocitrate dehydrogenase [NADP] | AKA> | **NC** | No |
|  | 6PGDH | At3g02360 | Phosphogluconate dehydrogenase | SKI> | C | 1D | Y | Y | Y | Y | XP_004238989.1 | 6-phosphogluconate dehydrogenase, decarboxylating 3 | SKI> | C | Yes |
|  | BADH | At3g48170 | Aldehyde dehydrogenase | SKL> | C | 3F | Y | Y | Y | Y | NP_001333606.1 | Putative betaine aldehyde dehyrogenase | SKN> | **NC** | No |
|  | HBCDH | At3g15290 | Hydroxybutyryl-CoA dehyrogenase | PRL> | **NC** |  | Y | Y | Y | Y | XP_010324368.1 | Uncharacterized protein LOC101257879 | PRL> | **NC** | No |
|  | ZnDH | At3g56460 | Zinc-binding dehydrogenase | SKL> | C | 2F | Y | Y | Y | Y | XP_004235213.1 | Quinone oxidoreductase-like protein 2 homolog | SKL> | C | Yes |
|  | GAPC2 | At1g13440 | Glyceraldehyde 3-phosphate dehydrogenase C2 | SKA> | **NC** | 3F | x | x | Y | x | NP_001266254.2 | Glyceraldehyde-3-phosphate dehydrogenase | AKA> | **NC** | No |
| Uncharacterized Proteins | UP9 | At1g29120 | Unknown protein 9 | ASL> | **NC** | 3F | x | x | Y | x | XP_004233040.1 | Putative lipase YDR444W isoform X1 | PSL> | n | No |
|  | UP7 | At5g65400 | Unknown protein 7 | SLM> | **NC** | 2F | x | Y | x | Y | XP_004250132.1 | Esterase AGAP003155 | STV> | n | No |
|  | UP3 | At2g31670 | Unknown protein 3 | SSL> | **NC** | 3F | x | Y | Y | Y | XP_010324012.1 | Stress-response A/B barrel domain-containing protein UP3 | ASL> | **NC** | No |
|  | UP6 | At1g16730 | Unknown protein 6 | SKL> | C | 2F | x | Y | Y | Y | - | No ortholog found | - | - | - |
|  | UP5 | At5g44250 | Unknown protein 5 | SRL> | C | 2F | x | Y | x | Y | XP_004238167.1 | Uncharacterized protein LOC101257658 | SRL> | C | Yes |
| Oxidases | CuAO | At2g42490 | Copper amine oxidase | SKL> | C | 2F | Y | Y | Y | Y | XP_004239124.1 | Uncharacterized protein LOC101266123 | SKL> | C | Yes |
|  | PAO4 | At1g65840 | Polyamine oxidase 4 | SRM> | C |  | Y | x | x | Y | XP_004234492.1 | Probable polyamine oxidase 4 | SRM> | C | Yes |
|  | PAO2 | At2g43020 | Polyamine oxidase 2 | SRL> | C |  | x | x | x | Y | XP_004243630.1 | Probable polyamine oxidase 2 | SRM> | C | Yes |
|  | PAO3 | At3g59050 | Polyamine oxidase 3 | SRM> | C |  | x | x | x | Y | XP_004251556.1 | Probable polyamine oxidase 2 | SRM> | C | Yes |
|  | SO | At3g01910 | Sulfite oxidase | SNL> | **NC** |  | Y | Y | Y | Y | ABI53846.1 | Sulfite oxidase | ANL> | **NC** | Yes |
|  | ACX1 | At4g16760 | Acyl-CoA oxidase 1 | ARL> | C |  | Y | Y | Y | Y | NP_001234198.1 | Peroxisomal acyl-CoA oxidase 1A | AKL> | C | Yes |
|  | ACX5 | At2g35690 | Acyl-CoA oxidase 5 | AKL> | C |  | Y | Y | x | Y | NP_001234198.1 | Peroxisomal acyl-CoA oxidase 1A | AKL> | C | Yes |
|  | ACX4 | At3g51840 | Acyl-CoA oxidase 4 | SRL> | C |  | Y | Y | Y | Y | XP_004249072.1 | Acyl-coenzyme A oxidase 4, peroxisomal | SRL> | C | No |
|  | HAOX1 | At3g14150 | Hydroxy-acid oxidase 1 | SML> | **NC** | 2F |  | Y |  | Y | XP_004235559.1 | Peroxisomal (S)-2-hydroxy-acid oxidase GLO4 isoform X1 | CRM> | **NC** | No |
|  | GO1 | At3g14415 | Glycolate oxidase 1 | PRL> | **NC** |  | Y | Y | Y | Y | NP_001294871.1 | Peroxisomal (S)-2-hydroxy-acid oxidase GLO1 | PRL> | **NC** | No |
|  | GO2 | At3g14420 | Glycolate oxidase 2 | ARL> | C |  | Y | Y | Y | Y | NP_001294871.1 | Peroxisomal (S)-2-hydroxy-acid oxidase GLO1 | PRL> | **NC** | No |
|  | GO3 | At4g18360 | Glycolate oxidase 3 | AKL> | C | 3F | Y | Y | Y | Y | XP_019070919.1 | (S)-2-hydroxy-acid oxidase GLO1 isoform X1 | PRL> | **NC** | No |
| Phosphatases and Kinases Family | pxPfkB | At1g49350 | PfkB-type carbohydrate kinase family protein | SML> | **NC** |  | Y | x | x | Y | XP_004230905.1 | uncharacterized protein LOC101251824 | SKL> | C | Yes |
|  | GPK1 | At3g17420 | Glyoxysomal protein kinase1 | AKI> | C |  | x | x | x | Y | XP_010313851.1 | Probable receptor-like protein kinase At5g18500 | HQV> | n | No |
|  | NADK3 | At1g78590 | NADH Kinase 3 | SRY> | **NC** |  | x | x | x | Y | XP_010324401.1 | NADH kinase isoform X1 | VVA> | n | No |
|  | PAP7 | At2g01880 | purple acid phosphatase 7 | AHL> | **NC** |  | x | x | x | X | XP_004236915.1 | Purple acid phosphatase 4 | SNI> | **NC** | No |
|  | ECI | At1g65520 | Monofunctional enoyl CoA | SKL> | C |  | Y | Y | x | Y | XP_004229827.1 | Enoyl-CoA delta isomerase 1, peroxisomal | AKL> | C | No |
|  | ECH2 | At1g76150 | Monofunctional enoyl-Co A hydratase 2 | SSL> | **NC** |  | Y | Y | Y | Y | XP_004253134.1 | Enoyl-CoA hydratase 2, peroxisomal | SSL> | **NC** | Yes |
|  | ATF1 | At1g21770 | Acteyl transferase 1 | SSI> | **NC** |  | x | Y | x | Y | XP_004245245.1 | Acetyltransferase At1g77540 | SHI> | **NC** | No |
|  | ATF2 | At1g77540 | Acetyltransferase | SSI> | **NC** | 2F | x | Y | Y | Y | XP_004245245.1 | Acetyltransferase At1g77540 | SHI> | **NC** | No |
|  | Uri | At2g26230 | Uricase | SKL> | C |  | Y | Y | Y | Y | NP_001234208.1 | Urate oxidase | SKM> | C | Yes |
|  | GLH | At2g38180 | GDSL motif lipase/hydrolase family protein | ARL> | C |  | Y | x | x | Y | XP_004240943.1 | GDSL esterase/lipase CPRD49 | QWD> | n | No |
|  | EH3 | At4g02340 | Epoxide hydrolase 3 | ASL> | **NC** |  | Y | Y | x | Y | XP_004240550.1 | uncharacterized protein LOC101260234 | QKF> | n | No |
|  | NUDT19 | At5g20070 | Nudix hydrolase homolog 19 | SSL> | **NC** |  | x | x | x | Y | XP_004241279.1 | Nudix hydrolase 19, chloroplastic isoform X1 | SNL> | **NC** | No |
|  | MFP2 | At3g06860 | Fatty acid multifuncional protein 2 | SRL> | C |  | Y | Y | Y | Y | XP_004252912.1 | Glyoxysomal fatty acid beta-oxidation multifunctional protein MFP-a isoform X1 | SRL> | C | Yes |
|  | SCO3 | At3g19570 | Snowy Cotyledon 3 | SRL> | C |  | x | x | x | Y | XP_004230707.1 | QWRF motif-containing protein 2-like | STT> | n | No |
|  | ICL | At3g21720 | Isocitrate lyase | SRM> | C |  | x | x | Y | Y | NP_001233878.2 | Isocitrate lyase | ARM> | C | No |
|  | MIF | At3g51660 | Macrophage migration inhibitory factor | SKL> | C |  | x | Y | x | Y | XP_004249685.1 | Macrophage migration inhibitory factor homolog | SKL> | C | Yes |
|  | MCD | At4g04320 | Malonyl-CoA decarboxylase | SRL> | C | 2F | Y | Y | x | Y | XP_025884994.1 | Malonyl-CoA decarboxylase, mitochondrial | IFT> | n | Yes |
|  | IBR3 | At3g06810 | IBA-response 3 | SKL> | C |  | Y | Y | Y | Y | XP_004239801.1 | Probable acyl-CoA dehydrogenase IBR3 | SRL> | C | No |
|  | IBR1 | At4g05530 | Indole-3-butyric acid response 1 | SRL> | C | 1D | Y | Y | Y | Y | XP_004236675.1 | Tropinone reductase-like 3 | SRL> | C | Yes |
|  | IBR10 | At4g14430 | Indole-3-butyric acid response 10 | PKL> | **NC** | 1D | Y | Y | Y | Y | XP_004241014.1 | Enoyl-CoA delta isomerase 2, peroxisomal | SHL> | **NC** | No |
|  | ECHIA | At4g16210 | Monofunctional enoyl-CoA hydratse/isomerase a | SKL> | C | 1D | Y | Y | Y | Y | XP_004229012.1 | Probable enoyl-CoA hydratase 1, peroxisomal | SKL> | C | Yes |
|  | AIM1 | At4g29010 | Abnormal inflorescence meristem 1 | SKL> | C |  | Y | Y | Y | Y | XP_004242934.1 | Peroxisomal fatty acid beta-oxidation multifunctional protein AIM1 | SRM> | C | Yes |
|  | MLS | At5g03860 | Malate synthase | SRL> | C |  | x | x | Y | Y | XP_004236345.1 | Malate synthase, glyoxysomal | SRL> | C | Yes |
|  | BIOTIN F | At5g04620 | 7-Keto-8-aminopelargonic acid synthase | PKL> | **NC** |  | x | x | x | Y | XP_004240762.1 | 8-amino-7-oxononanoate synthase isoform X1 | ARL> | C | No |
|  | CSD3 | At5g18100 | Copper/zinc superoxide dismutase 3 | AKL> | C |  | x | Y | Y | Y | XP_004234809.1 | Superoxide dismutase [Cu-Zn] 2 isoform X1 | SSV> | n | No |
|  | MIA40 | At5g23395 | Mitochondrial intermembrane space assembly machinery 40 | SKL> | C |  | x | x | x | Y | XP_004236875.1 | Mitochondrial intermembrane space import and assembly protein 40 homolog | TKL> | **NC** | Yes |
|  | 6PGL | At5g24400 | 6-Phosphogluconolactonase | SKL> | C |  | x | x | x | x | XP_025887973.1 | Probable 6-phosphogluconolactonase 4, chloroplastic isoform X1 | SKL> | C | Yes |
|  | AtHsp15.7 | At5g37670 | Heat shock protein similar to 17.6kDa class 1 | SKL> | C |  | x | x | Y | Y | XP_004236689.1 | 15.7 kDa heat shock protein, peroxisomal | SKL> | C | Yes |
|  | GSTT1 | At5g41210 | Glutathione S-transferase θ isoform 1 | SKI> | C |  | Y | Y | Y | Y | XP_004245615.1 | Glutathione S-transferase T1 | SKM> | C | Yes |
|  | SCP2 | At5g42890 | Sterol carrier protein 2 | SKL> | C |  | Y | Y | Y | Y | XP_004238156.1 | Non-specific lipid-transfer protein-like 1 | SKM> | C | Yes |
|  | AtDCI | At5g43280 | Δ3,5-Δ2,4-Enoyl-CoA-isomerase | AKL> | C |  | Y | Y | Y | Y | XP_004239303.1 | Delta(3,5)-Delta(2,4)-dienoyl-CoA isomerase, peroxisomal | AKL> | C | Yes |
|  | ACAT1.3 | At5g47720 | Acetoacetyl-CoA thiolase 1.3 | SAL> | **NC** |  | x | x | Y | Y | XP_004236650.1 | Probable acetyl-CoA acetyltransferase, cytosolic 2 | SNL> | **NC** | Yes |
|  |  |  |  |  |  |  |  |  |  |  |  |  |  |  |  |
|  | BZO1 | At1g65880 | Benzoyloxyglucosinolate 1 | SRL> | C | 3F | x | x | Y | x | XP_004234491.1 | Butyrate--CoA ligase AAE11, peroxisomal-like | SRL> | C | Yes |
|  | ANNAt3 | At2g38760 | annexin D3 isoform X2 | SKI> | C |  | x | x | x | x | XP_004230105.1 | Annexin D3 isoform X2 | AKV> | **NC** | No |
| **Peroxisomal Protein without Obvious PTS** | | | | | | | | | | | **Putative Peroxisomal Protein without Obvious PTS** | | | | |
|  | B12D1 | At3g48140 | Senescence-associated protein/B12D-related protein | PTY> | n | 2F | Y | x | x | x | XP_004232259.1 | Uncharacterized protein LOC101265497 | NPN> | n | No |
|  | HIT1 | At4g16566 | Histidine traid family protein 1 | AT*S*> | n | 2F | Y | Y | Y | Y | XP_004244571.1 | Bifunctional adenosine 5'-phosphosulfate phosphorylase/adenylylsulfatase HINT4 | SSM> | **NC** | Yes |
|  | ATMS1 | At5g17920 | Cobalamin independent methionine synthase | SAK> | n | 3F | x | Y | Y | Y | NP_001306983.1 | 5-methyltetrahydropteroyltriglutamate--homocysteine methyltransferase | SAK> | n | No |
| Phosphatases and Kinases Family | NDPK1 | At4g09320 | Nucleoside diphosphate kinase type 1 | YET> | n | 2F | x | Y | Y | x | NP_001234174.2 | Nucleoside diphosphate kinase | IYE> | n | No |
|  | CoAE | At2g27490 | Dephospho-CoA kinase | IGS> | n | 2F | x | Y | x | x | XP_004250350.1 | uncharacterized protein LOC101256544 | RSP> | n | No |
